# Long-Term Training with a Brain-Machine Interface-Based Gait Protocol Induces Partial Neurological Recovery in Paraplegic Patients

**DOI:** 10.1038/srep30383

**Published:** 2016-08-11

**Authors:** Ana R. C. Donati, Solaiman Shokur, Edgard Morya, Debora S. F. Campos, Renan C. Moioli, Claudia M. Gitti, Patricia B. Augusto, Sandra Tripodi, Cristhiane G. Pires, Gislaine A. Pereira, Fabricio L. Brasil, Simone Gallo, Anthony A. Lin, Angelo K. Takigami, Maria A. Aratanha, Sanjay Joshi, Hannes Bleuler, Gordon Cheng, Alan Rudolph, Miguel A. L. Nicolelis

**Affiliations:** 1 Neurorehabilitation Laboratory, Associação Alberto Santos Dumont para Apoio à Pesquisa (AASDAP), Sâo Paulo, Brazil; 2 Associação de Assistência à Criança Deficiente (AACD), São Paulo, Brazil; 3 Edmond and Lily Safra International Institute of Neuroscience, Santos Dumont Institute, Macaiba, Brazil; 4 Alberto Santos Dumont Education and Research Institute, Sao Paulo, Brazil; 5 STI IMT, Ecole Polytechnique Federal de Lausanne, Lausanne, Switzerland; 6 Department of Biomedical Engineering, Duke University, Durham, NC, USA; 7 Mechanical and Aerospace Engineering, University of California, Davis, CA, USA; 8 Institute for Cognitive Systems, Technical University of Munich (TUM), Munich, Germany, Germany; 9 Colorado State University, Fort Collins, CO, USA; 10 Department of Neurobiology, Duke University, Durham, NC, USA; 11 Department of Psychology and Neuroscience, Duke University, Durham, NC, USA; 12 Center for Neuroengineering, Duke University, Durham, NC, USA

## Abstract

Brain-machine interfaces (BMIs) provide a new assistive strategy aimed at restoring mobility in severely paralyzed patients. Yet, no study in animals or in human subjects has indicated that long-term BMI training could induce any type of clinical recovery. Eight chronic (3–13 years) spinal cord injury (SCI) paraplegics were subjected to long-term training (12 months) with a multi-stage BMI-based gait neurorehabilitation paradigm aimed at restoring locomotion. This paradigm combined intense immersive virtual reality training, enriched visual-tactile feedback, and walking with two EEG-controlled robotic actuators, including a custom-designed lower limb exoskeleton capable of delivering tactile feedback to subjects. Following 12 months of training with this paradigm, all eight patients experienced neurological improvements in somatic sensation (pain localization, fine/crude touch, and proprioceptive sensing) in multiple dermatomes. Patients also regained voluntary motor control in key muscles below the SCI level, as measured by EMGs, resulting in marked improvement in their walking index. As a result, 50% of these patients were upgraded to an incomplete paraplegia classification. Neurological recovery was paralleled by the reemergence of lower limb motor imagery at cortical level. We hypothesize that this unprecedented neurological recovery results from both cortical and spinal cord plasticity triggered by long-term BMI usage.

Spinal Cord Injury (SCI) rehabilitation remains a major clinical challenge, especially in cases involving chronic complete injury. Clinical studies using body weight support systems^1^,^2^, robotic assistance^1^,^2^,^3^,^4^, and functional electrostimulation of the leg^5^,^6^ have proposed potential solutions for assisting SCI patients in walking^7^,^8^. Yet, none of these approaches have generated any consistent clinical improvement in neurological functions, namely somatosensory (tactile, proprioceptive, pain, and temperature) perception and voluntary motor control, below the level of the spinal cord lesion.

Since the first experimental demonstrations in rats^9^, monkeys^10^,^11^, and the subsequent clinical reports in humans^12^,^13^,^14^, brain-machine interfaces (BMIs) have emerged as potential options to restore mobility in patients who are severely paralyzed as a result of spinal cord injuries (SCIs) or neurodegenerative disorders^15^. However, to our knowledge, no study has suggested that long-term training associating BMI-based paradigms and physical training could trigger neurological recovery, particularly in patients clinically diagnosed as having a complete SCI. Yet, in 60–80% of these “complete” SCI patients, neurophysiological assessments^16^,^17^ and post-mortem anatomical^18^ studies have indicated the existence of a number of viable axons crossing the level of the SCI. This led some authors to refer to these patients as having a “discomplete” SCI^17^ and predict that these remaining axons could mediate some degree of neurological recovery.

For the past few years, our multidisciplinary team has been engaged in a project to implement a multi-stage neurorehabilitation protocol – the Walk Again Neurorehabilitation (WA-NR) – in chronic SCI patients. This protocol included the intensive employment of immersive virtual-reality environments, combining training on non-invasive brain-control of virtual avatar bodies with rich visual and tactile feedback, and the use of closed-loop BMI platforms in conjunction with lower limb robotic actuators, such as a commercially available robotic walker (Lokomat, Hocoma AG, Volketswil, Switzerland), and a brain-controlled robotic exoskeleton, custom-designed specifically for the execution of this project.

Originally, our central goal was to explore how much such a long-term BMI-based protocol could help SCI patients regain their ability to walk autonomously using our brain-controlled exoskeleton. Among other innovations, this device provides tactile feedback to subjects through the combination of multiple force-sensors, applied to key locations of the exoskeleton, such as the plantar surface of the feet, and a multi-channel haptic display, applied to the patient’s forearm skin surface.

Unexpectedly, at the end of the first 12 months of training with the WA-NR protocol, a comprehensive neurological examination revealed that all of our eight patients had experienced a significant clinical improvement in their ability to perceive somatic sensations and exert voluntary motor control in dermatomes located below the original SCI. EEG analysis revealed clear signs of cortical functional plasticity, at the level of the primary somatosensory and motor cortical areas, during the same period. These findings suggest, for the first time, that long-term exposure to BMI-based protocols enriched with tactile feedback and combined with robotic gait training may induce cortical and subcortical plasticity capable of triggering partial neurological recovery even in patients originally diagnosed with a chronic complete spinal cord injury.

## Methods

**Figure 1:**
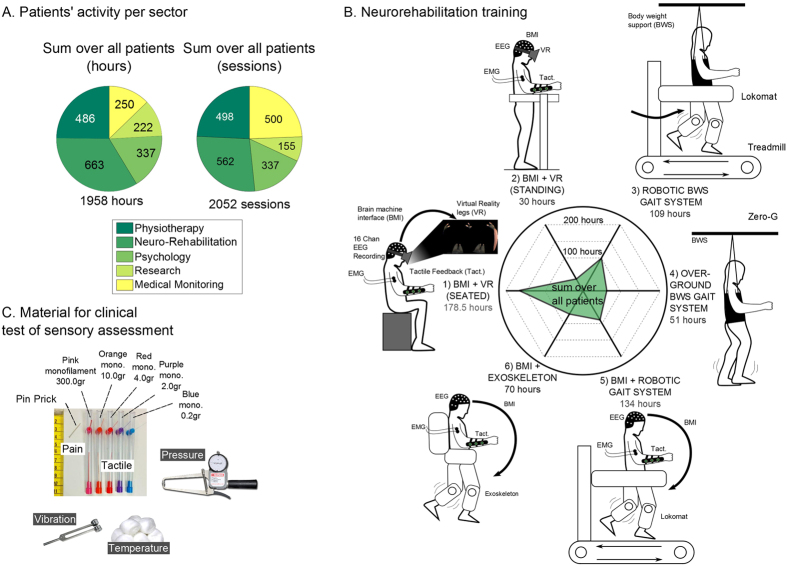
Methodology. **(A**) Cumulated number of hours and sessions for all patients over 12 months. We report cumulated hours for the following activities: classic physiotherapy activities (e.g. strengthening/stretching), gait-BMI-based neurorehabilitation, one-to-one consultations with a psychologist, periodic measurements for research purposes and routine medical monitoring (vital signs, etc.). (**B**) Neurorehabilitation training paradigm and corresponding cumulated number of hours for all patients: 1) Brain controlled 3D avatar with tactile feedback when patient is seated on a wheelchair or 2) in an orthostatic position on a stand-in-table, 3) Gait training using a robotic body weight support (BWS) system on a treadmill (LokomatPro, Hocoma), 4) Gait training using an overground BWS system (ZeroG, Aretech). 5–6) Brain controlled robotic gait training integrated with the sensory support of the tactile feedback at gait devices (BWS system on a treadmill or the exoskeleton). (**C**) Material used for the clinical sensory assessment of dermatomes in the trunk and lower limbs: to evaluate pain sensitivity, examiner used a pin-prick in random positions of the body segments. Nylon monofilaments applying forces ranging between 300 to 0.2 grams on the skin, were used to evaluate patients’ sensitivity for crude to fine touch. Dry cotton and alcohol swabs were used to assess respectively warm and cold sensation. Vibration test was done using a diapason on patients’ legs bone surface. Deep pressure was assessed with an adapted plicometer in every dermatome.

Eight paraplegic patients, suffering from chronic (>1 year) spinal cord injury (SCI, seven complete and one incomplete, see Fig. 1A, Supplementary Methods Inclusion/exclusion Criteria), were followed by a multidisciplinary rehabilitation team, comprised of clinical staff, engineers, neuroscientists, and roboticists, during the 12 months of 2014. Our clinical protocol, which we named the Walk Again Neurorehabilitation (WA-NR), was approved by both a local ethics committee (Associação de Assistência à Criança Deficiente, Sao Paulo, Sao Paulo, Brazil #364.027) and the Brazilian federal government ethics committee (CONEP, CAAE: 13165913.1.0000.0085). All research activities were carried out in accordance with the guidelines and regulations of the Associação de Assistência à Criança Deficiente and CONEP. Each participant signed written informed consent before enrolling in the study. The central goal of this study was to investigate the clinical impact of the WA-NR, which consisted of the integration between traditional physical rehabilitation and the use of multiple brain-machine interface paradigms (BMI). This protocol included six components: (1) an immersive virtual reality environment in which a seated patient employed his/her brain activity, recorded via a 16-channel EEG, to control the movements of a human body avatar, while receiving visuo-tactile feedback; (2) identical interaction with the same virtual environment and BMI protocol while patients were upright, supported by a stand-in-table device; (3) training on a robotic body weight support (BWS) gait system on a treadmill (Lokomat, Hocoma AG, Switzerland); (4) training with a BWS gait system fixed on an overground track (ZeroG, Aretech LLC., Ashburn, VA); (5) training with a brain-controlled robotic BWS gait system on a treadmill; and (6) gait training with a brain-controlled, sensorized 12 degrees of freedom robotic exoskeleton (see Supplementary Material).

In all cases except components 3 and 4 above, patients received continuous streams of tactile feedback from either the virtual (body avatar) or robotic devices (Lokomat and exoskeleton) via a haptic display (consisting of arrays of coined shaped vibrators) applied to the skin surface of the patient’s forearms. Tactile stimulation on the forearm was given in accordance with the rolling of the ipsilateral virtual or robotic feet on the ground.

Two BMI strategies were employed throughout training. Initially, patients were required to imagine movement of the arms to modulate EEG activity so that they could generate high level motor commands such as ‘walk’ or ‘stop’. Once patients mastered this first method, they learned to use EEG signals to control individual avatar/robotic leg stepping by imagining movements of their own legs (see Supplementary Material for details). For the first paradigm, after the selection of the correct state, patients confirmed their choice by performing an isometric contraction of the triceps muscle.

Three lower limb actuators were used for the BMI experiments: a simulated 3D virtual avatar, the Lokomat gait trainer (Hocoma AG, Switzerland); and a custom built exoskeleton. The virtual avatar was simulated in MotionBuilder (Autodesk MotionBuilder 2014) and visualized from first person perspective using an immersive head mounted displayer (Oculus Rift, Oculus VR). The Lokomat is a robotic gait device that uses a body weight support (BWS) system integrated with a treadmill. Finally, a custom built exoskeleton was used with our patients. The exoskeleton had autonomous power, self-stabilization, and full lower limb hydraulic actuation. It was built to accommodate a wide weight range of SCI patients (50–80 kg) while not necessitating the use of crutches (See Supplementary Movie S11). Our exoskeleton was used in conjunction with a ZeroG^19^ system containing an overground BWS system that rides along an overhead fixed track. In this setup, there were no mechanical barriers between the patient and physical therapist. For this reason, the walking setup offers more challenges to the subjects, in comparison to off-the-shelf devices, by requiring patients to be in charge of postural and trunk control, upper limb strength and dynamic balance. Further gait training was performed by having subjects utilize a lower limb orthosis and walking assistive devices (hip-knee-ankle-foot orthosis or ankle-foot orthosis with knee extension splint and wheeled triangular walker).

Throughout the application of our protocol, the complexity of activities was increased over time to ensure cardiovascular system stability and better patient postural control; starting with orthostatic training at a stand-in-table and progressing all the way to the different gait training robotic systems^20^,^21^,^22^. In addition to routine general clinical evaluations (i.e. cardiovascular function, intestinal and urinary emptying, skin inspection, spasticity handling), before and after every activity, and a long-term treatment of osteoporosis, multiple clinical evaluations were periodically performed in order to identify possible changes in the neurological status of the SCI and to assess psychological and physical conditions. Such clinical evaluation started on the first day patients began training (Day 0), and were repeated after 4, 7, 10, and 12 months. Clinical evaluations included: the American Spinal Injury Association (ASIA) Impairment Scale (International Standards for the Neurological Classification of Spinal Cord Injury^23^), the Semmes-Weinstein Monofilament Test^24^ (Fig. 1B), the evaluation of temperature, vibration, proprioception and deep pressure sensitivity, a muscle strength test (Lokomat L-force Evaluation)^25^,^26^, the Thoracic-Lumbar Scale for trunk control assessment^27^, Walking Index Spinal Cord Injury II (WISCI)^28^, Spinal Cord Independence Measurement III (SCIM)^29^, McGill Pain Questionnaire^30^ and Visual Analogue Scale (VAS)^31^,^32^ for pain evaluation, the range of motion of lower limb joints of the Medical Research Council scale^33^, Modified Ashworth Scale^34^ and the Lokomat L-stiff Evaluation for spasticity^35^, the World Health Organization Quality of Life Assessment Instrument-Bref (WHOQoL-Bref)^36^, the Rosenberg Self-Esteem Scale^37^, and the Beck Depression Inventory (BDI)^38^.

Throughout training, the potential occurrence of functional cortical plasticity was evaluated through longitudinal analyses of EEG recordings. For this, patients were instructed to imagine movements of their own legs while EEG signals from 11 scalp electrodes were recorded over the leg primary somatosensory and motor cortical areas. Independent Component Analysis (ICA,^39^) was employed to determine potential cortical sources, represented by individual independent components (ICs), of novel leg representations in the primary motor and somatosensory cortices and to detect functional changes of these representations over time. To evaluate brain dynamics modulation, before and after many months of training, we calculated for each IC the Event Related Spectral Perturbations (ERSPs) with respect to a baseline of 1 second prior to the event and normalized by the average power across trials at each frequency. Event Related Potentials (ERPs), sampled from two EEG electrodes located over the leg representation area, averaged over all patients, before and after training, were also calculated and used for statistical comparison.

## Results

Altogether, the eight paraplegic patients enrolled in this protocol (Table 1 for patient’s demography) participated in a total of 2,052 sessions, for a collective total of 1,958 hours, divided into multiple phases of neurorehabilitation training (Fig. 1A). Figure 1B details the distribution of hours of the six stages employed in our WA-NR protocol.

**Figure 2:**
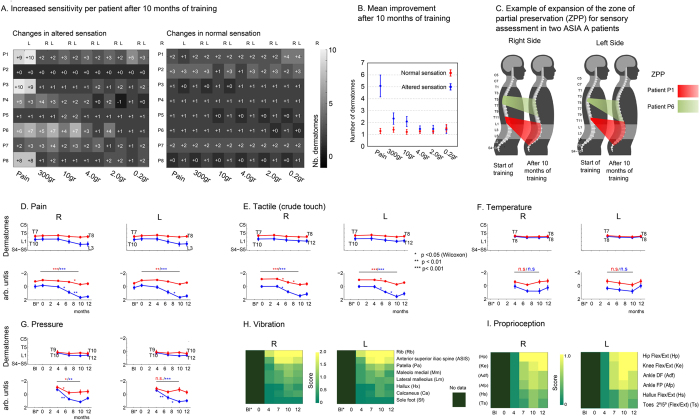
Sensory improvement after neurorehabilitation training. **(A)** Left table reports the improvement for altered sensation (hyper or hypoesthesia) and the right table reports normal sensation. Number of dermatomes recovered after 10 months training compared to baseline (recorded at day 1 of training) for pain and tactile sensory modalities for all patients and body sides. Sensory modalities are reported by pain, crude touch (applied with a 300 gr. monofilament), and gradually more selective touch (applied with 10 gr. to 0.2 gr. monofilaments). The brightness of each colored square represents the magnitude of improvement, considering each monofilament and pin employed (lightest represents highest improvement). (**B**) Average sensory improvement (mean +/− SEM over all patients) after 10 months training. (**C**) Example of improvement in the Zone of Partial Preservation for sensory evaluation for two patients. (**D–G**) Mean +/− SEM of lowest dermatome with normal (red) or altered (blue) sensation for (**D**) Pain (**E**) Tactile – crude touch, pink monofilament (**F**) Temperature and (**G**) Pressure on the body calculated over all patients. From (**D**–**G**), y-axis exhibits dermatomes in a cranio-caudal order, following the anatomic sequence. Baseline was recorded during the year following the injury, time 0 represents the starting day of our training. For each modality we show the average over raw (top graph) and z-scored data (lower graph). P-values for Wilcoxon rank sum test are reported on z-scored data (*p < 0.05, **p < 0.01, ***p < 0.001). (**H)** Mean score for perception of vibration on eight leg bones presented (most proximal to most distal order). Score convention was the following: 0 for no sensation, 1 for altered sensation and 2 for normal sensation. (**I**) Mean score for proprioception (0: absent, 1: present) over lower limb joints. Note that measurements for temperature, vibration and proprioception were introduced 4 months after the beginning of the training.

As part of the neurological evaluation, we periodically tested all patients’ sensitivity to fine touch, pain, temperature, vibration, pressure, and proprioception (Fig. 1C). Figure 2A describes the individual improvement of each patient, in number of dermatomes, for tactile (Semmes-Weinstein monofilament test) and pain sensitivity (ASIA sensory evaluation) for all eight patients at the end of 10^th^ month of training. The left graph shows the improvement of normal sensation, while the right graph depicts the improvement in altered sensation (hyper or hypoesthesia). In each of the graphs, improvement is described in terms of number of dermatomes, i.e. the body area that receives sensory innervation from a given spinal nerve root. In this analysis, we measured the extent of the Zone of Partial Preservation (ZPP), i.e. the dermatomes and myotomes (the set of muscles innervated by one spinal nerve) caudal to the neurological level of injury that remain partially innervated^23^. The ZPP applies only for complete SCI patients.

Figure 2B displays the patients’ average improvement, in number of dermatomes below the SCI, for both tactile and pain sensation after 10 months of training. Inspection of these two figures reveals significant improvement in every monofilament and pin tested for all patients. Although we observed significant improvements in both the zone of normal sensation and in the ZPP, the largest gains (5.1 +/− 0.9 dermatomes) were consistently observed in the latter in every patient. In terms of tactile sensitivity, the largest improvements in number of dermatomes (1 normal/2 altered both pink and orange) were observed for the pink (300 g) and orange monofilaments (10 g). For the orange monofilament, the improvement was 1 dermatome for normal sensory area and 2 dermatomes for the ZPP area. Figure 2C depicts two of the patients who exhibited the best improvement in both fine tactile and nociceptive perception. Notice that the improvement was larger for the latter and includes many dermatomes below the SCI level.

Throughout the study, all patients reported some type of pain sensation below the SCI level, as measured by the McGill’s questionnaire. Yet, patients experienced some difficulty in reporting the exact location of such pain. This finding corroborates the results described by Demirel *et al*.^40^ who showed that 68% of their SCI patients reported pain in the lower limbs. As the training progressed, the patients’ self-reported pain intensity in daily life - as measured by the visual analogue scale (VAS,^32^) - decreased on average from 2.0 to 1.3 (maximum 10, n = 8) while perception of pain at the moment of the evaluation (Present Pain Intensity evaluation integrates McGill Questionnaire^30^) stayed very low (sum over all patients = 3, with a maximum possible score = 40) throughout the year. An improvement in patients’ ability to perceive and relate more effectively the occurrence of lower limb pain as well the pain location in the body was observed at the end of the study. Supplementary Movie S1 shows the mean improvement for pain sensation over the 1 year training period.

Figure 2D–I describes the mean evolution of every somatosensory parameter (pain, tactile, temperature, pressure, vibration, and proprioception) measured over the time course of our study. These graphs revealed that we did not observe any significant improvement in temperature sensitivity (Fig. 2F). Conversely, pressure sensitivity improved by 1 (normal) and 2 (altered) dermatomes, between the 4^th^ and 10^th^ month (Fig. 2G). Figure 2H indicates that the average sensitivity to vibration also improved, mainly for the hip joint (anterior superior iliac spine), but also for the knee and the ankle. In all patients, improvement went from almost total absence to mid-level vibration sensation (see Figure S1 for details per patient).

The average proprioception sensation across all patients also improved significantly during the period between the 4^th^–12^th^ months (Fig. 2I). This improvement was mainly observed at the hip level, since patients became capable of distinguishing hip flexion and extension for both the right and left sides. Secondary improvement was detected at the knee and ankle, from the 7^th^ month to the 12^th^ month (see Figure S2 for details per patient).

**Figure 3:**
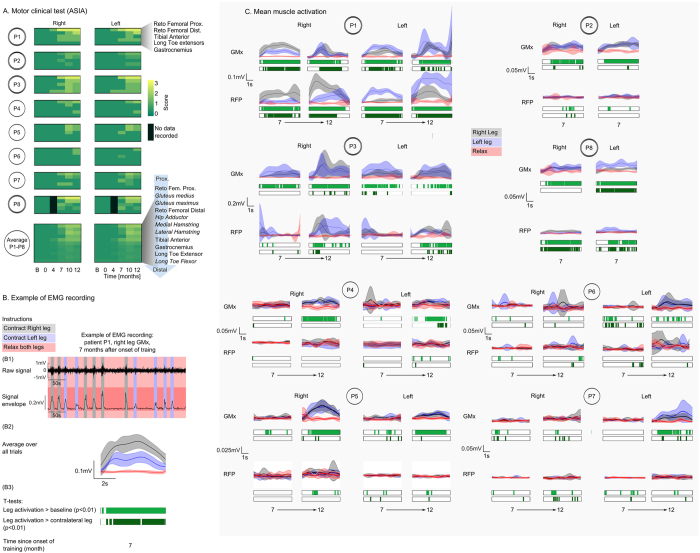
Clinical and neurophysiological assessments of lower limb motor recovery. **(A)** Detail of key muscle improvement per patient, according to clinical evaluation (ASIA) for all patients and the average over all 8 patients for key and secondary lower limb muscles listed in a proximal to distal order (secondary muscles are in italic). Patients that changed classification to ASIA C after 12 months of training have 2 lines rings around their names. (**B**) Details of the EMG recording procedure in SCI patients. (B1) Raw EMG for the right gluteus maximus muscle for patient P1 is shown at the top of the topmost graph. The lower part of this graph depicts the envelope of the raw EMG, after the signal was rectified and low pass filtered at 3 HZ. Gray shaded areas represent periods where the patient was instructed to move the right leg, while the blue shaded areas indicate periods of left leg movement. Red areas indicate periods where patients were instructed to relax both legs. (B2) All trials over one session were averaged (mean +/− standard deviation envelopes are shown) and plotted as a function of instruction type (gray envelope = contract right leg; blue = contract left leg; red = relax both legs). (B3) Below the averaged EMG record, light green bars indicate instances in which the voluntary muscle contraction (right leg) was significantly different (t-test, p < 0.01) than the baseline (periods where she/he was instructed to relax both legs). Dark green bars depict periods in which there was a significant difference (p < 0.01) between muscle contraction in the right versus the left leg. (**C**) EMG envelops and t-tests for all recording sessions, involving 4 muscles, for all 8 patients: left and right gluteus maximus (GMx) and reto femoral proximal (RFP) muscles. Color convention and figure organization follows the one of panel B. Data was collected after 7 months of training for all patients and for all but patients P2 and P8 after 12 months.

Improvement in motor function was also extensively documented in our eight patients. Figure 3A depicts the evolution of the ASIA motor protocol assessment for all patients, considering five key lower limb muscles: hip flexors (rectus femoris proximal portion), knee extensors (rectus femoris distal portion), ankle dorsiflexors (tibialis anterior), ankle plantar flexors (gastrocnemius), and long toe extensors. This analysis revealed that every patient exhibited some degree of improvement in voluntary muscle contraction below the SCI level. Indeed, seven patients experienced an improvement, ranging from two to multiple key muscles below the SCI level. The bottom shelf of Figure 3A depicts the average improvement for all eight patients for 11 muscles (five key muscles and six secondary muscles: gluteus medius, gluteus maximus, hip adductor, medial and lateral hamstrings, and long toe flexor) ranged in a proximal to distal order. Overall, the strongest motor improvement was observed in the six most proximal muscles.

Motor recovery was also documented through multi-channel surface EMG recordings (Fig. 3B,C). For this evaluation, patients were instructed verbally by a physiotherapist to alternate movements of their left leg with movements of their right leg, and periods in which neither leg should be moved (Fig. 3B). The first EMG session was recorded after 7 months of training (all patients), then a second session was obtained at 12 months (all patients except P2 and P8). Since all patients were completely paralyzed below the level of injury, none of them exhibited any motor activity below the level of their SCI at the onset of the training. However, 7 months into training, EMG recordings revealed that all patients started to show signs of motor recovery, indicated by their ability to voluntarily control at least one muscle below the level of the SCI (Fig. 3C). By the 12^th^ month of training, this motor recovery had stabilized and, in most cases, improved significantly. Figure 3B,C shows that patient P1 exhibited the strongest and most consistent voluntary contractions (Fig. 3B,C, Supplementary Movies S3, S4, S8, S9) in both left and right gluteus maximus (GMx) and reto femoral proximal (RFP) muscles (contraction significance, t-test, p < 0.01, is indicated in light green under each graph). This patient’s motor control was clearly selective, i.e., a stronger contraction of the right GMx and RFP was observed when the patient was instructed to contract the right leg, while left GMx and RFP contractions were produced following the command to contract the left leg (see selectivity significance reported in dark green under each figure, t-test, p < 0.01). Patient P3 exhibited strong significant contractions in both left and right GMx muscles (Fig. 3C, Supplementary Movie S4). However, reliable RFP contractions, absent at the 7^th^ month, appeared and became consistent at the end of the 12^th^ month of training. The same evolution trend in motor control was observed in three other patients, P4, P5, and P7: while at the 7^th^ month these patients exhibited weak or no voluntary GMx muscle contractions, after 12 month of training such contractions became evident in the EMG recordings (bilateral for P4 and P5, left side in P7, Fig. 3C). Finally, patients P2 and P8 also displayed significant voluntary muscle contractions in their GMx muscles during the 7^th^ month of training. P8 also exhibited selective contractions in both her RPF muscles. Altogether, these longitudinal EMG recordings suggest that a sustained and long-term training protocol may be required to trigger motor improvement in ASIA A patients.

Motor contractions were also documented through the employment of the Lokomat torque sensors during the same type of task used for EMG measurements (Table 2). Patient P1 showed the best recovery at 21.14 N⋅m (12^th^ month, right leg) for hip flexion (thus RFP contraction), followed by patient P8 at 9.36 N⋅m (7^th^ month), and P3 and P5 at 4.6 and 4.3 N⋅m. Patients P2, P4, P6, P7 produced torques in the 0.8–1.5 N⋅m range.

**Figure 4:**
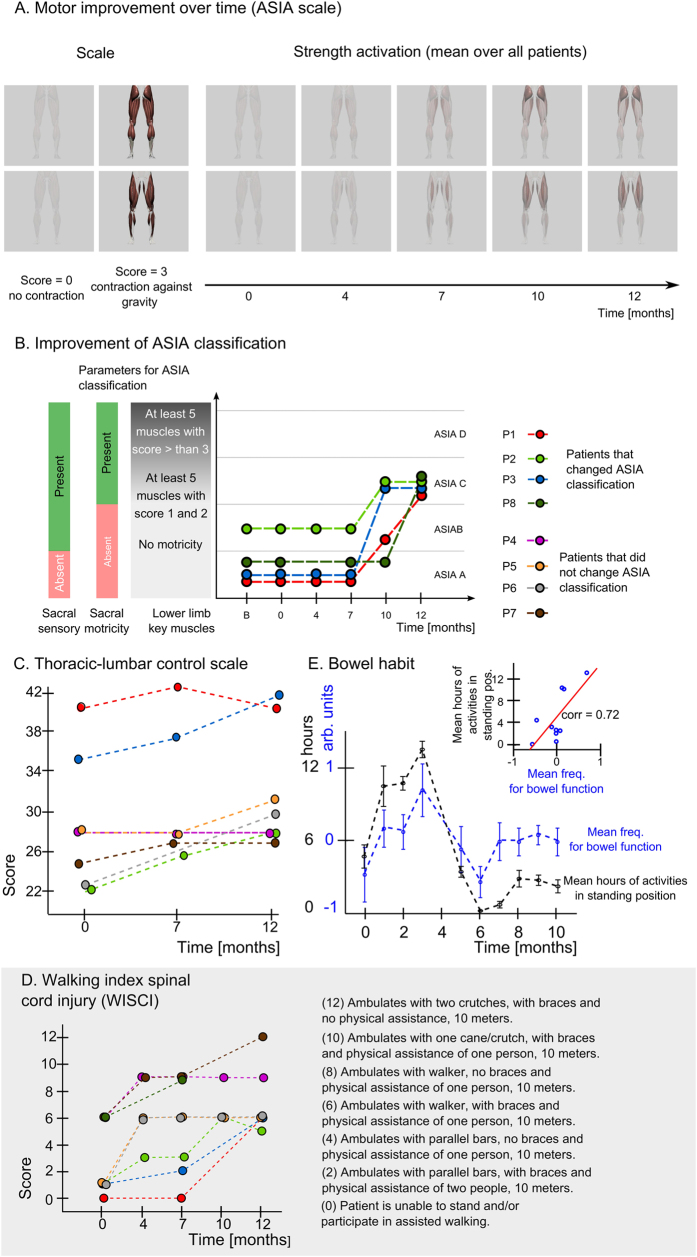
Clinical and functional improvements. (**A**) Graphical representation of clinical assessment of motor strength, calculated with the ASIA protocol, for key muscles and other muscles in the lower limb (mean over all patients). The scale for a given muscle goes from complete transparency for no muscle activation (muscle strength score 0) to contraction against gravity (score 3). Time 0 of x-axis means day 1 of the neuro-rehabilitation training. (**B**) Patients with ASIA classification improvements: four patients changed ASIA classification over the course of the neurorehabilitation training, three moved from ASIA A to C and one moved from ASIA B to C. ASIA A is characterized by absence of both motor and sensory functions in the lowest sacral area, ASIA B by the presence of sensory functions below the neurological level of injury, including sacral segments S4-S5 and no motor function is preserved more than three levels below the motor level on either side of the body, ASIA C by the presence of voluntary anal sphincter contraction, or sacral sensory sparing with sparing of motor function more than three levels below the motor level, majority of key muscles have muscle grade less than 3^23^. (**C**) Thoracic-lumbar control scale evaluates quantitatively motor skill of the thoracolumbar region. Score ranges between 0 and 65. It has 10 items that considers supine, prone, sitting and standing postures. In the present study, the last item (orthostatic position) was scored 0 due to the limitations of the pathology. (**D**) Functional assessment of autonomy in walking given by the Walking Index for Spinal Cord Injury scale. The scale ranges between 0, for a patient who is unable to stand and/or to participate in assisted walking, to 20 for a patient who ambulates 10 meters with no walking devices, no braces and no physical assistance. (**E**) Correlation between average time spent in a standing position in orthostatic or gait training (mean +/− SEM, values are average hours per month) and mean frequency for bowel function (values calculated per month and z-scored per patient).

Figure 4A and Supplementary Movie S2 illustrate the temporal progression of the average motor recovery observed in our patients, from month 0 to month 12, considering both key ASIA muscles and secondary lower limb muscles. Notice that this motor recovery clearly progressed from proximal to distal muscles, being more pronounced at the level of the hip joint. Thus, the muscles that exhibited the best recovery were: the gluteus maximum (maximum score 3; average score over all patients 1.56 and 1.5 for right and left side respectively), and gluteus medius (maximum score 2; average score 1.25 and 1.06), the proximal portion of the rectus femoris (max 3; average 1.56 and 1.5), and the hip adductors (max 2; average 1.18 and 1.06). At the level of the knee joint, the greatest improvements were observed in the medial and lateral hamstring (max 1; average 0.38 and 0.31), and the distal portion of the rectus femoris (max 2; average 1.06 and 0.88). At the ankle level, the greatest motor improvement was located in the sural triceps (max 1.5; average 0.31 and 0.25) and the anterior tibialis (max score 1; average 0.5 and 0.38).

Overall, the global patterns of both sensory and motor recovery indicated a proximal to distal progression, below the level of the patients’ SCI that evolved to include sacral roots. Supplementary Movies S3,S4,S5,S6,S7,S8,S9,S10 shows patients’ clear lower limb contractions while in a hanging or lying positions after 1 year of training with the WA-NR protocol. As a result of this sensory and motor recovery, Fig. 4B depicts the progression over time of the ASIA classification showing that 50% of our patients (n = 4) changed their ASIA classification in 12 months of training: three of these patients moved from ASIA A to ASIA C and one patient moved from ASIA B to ASIA C.

To further document this recovery, Fig. 4C displays the individual patient improvement in thoracic-lumbar strength and stability measured in different positions – seated and laying down – and static and dynamic balance. Between the 7^th^ and 10^th^ months, five of our patients improved significantly in this type of motor control. To illustrate how this motor recovery functionally impacted the patients, Fig. 4D depicts the progression of their Walking Index for SCI over the last 5 months of training. As one can see, all patients showed significant improvement in assisted walking skills; two patients increased their performance by six levels, four patients by five levels, and two more patients by three levels. For example, while Patient 1 was initially not even able to stand using braces when placed in an orthostatic posture (score 0), after 10 months of training the same patient became capable of walking using a walker, braces and the assistance of one therapist (score 6). At this stage, this patient became capable of producing voluntary leg movements mimicking walking, while suspended overground in the Lokomat (Supplementary Movie S8). The same patient produced close to 20N*m of hip flexion force while in the Lokomat (Table 2). In another example, Patient 7, started at score 6 and progressed all the way to score 12, which means that he/she was capable of walking with two crutches and lower limb orthoses (hip-knee-ankle-foot orthoses), while requiring no assistance by a therapist.

In addition to the partial recovery in neurological functions, our patients also exhibited improvements in gastrointestinal function and their overall skin condition. Figure 4E plots the monthly evolution of both the mean number of standing/walking hours and the z-scored mean frequency of bowel functioning. The top right graph in Fig. 4E shows that these latter two variables are highly correlated (r = 0.72, n = 10 measurements). Notice that peak bowel function was reached 3 months after the training starting. During the patient’s vacation period (months 4–5), lack of standing/walking was correlated with a significant reduction in bowel function. Upon restarting of the standing/walking training, bowel function increased again.

EEG measurements were employed to investigate potential cortical functional reorganization that could correlate with the type of sensorimotor improvement observed during training. An Independent Component Analysis (ICA^39^) was applied to 11 EEG channels both at the beginning, and after 8–10 months of training. Briefly, the ICA algorithm isolates maximally independent sources from multi-channel EEG signals to both discard non-brain signals (such as muscle artifact or noise) and identify the spatial location of distinct brain-derived signals contained in the overall EEG recording.

**Figure 5:**
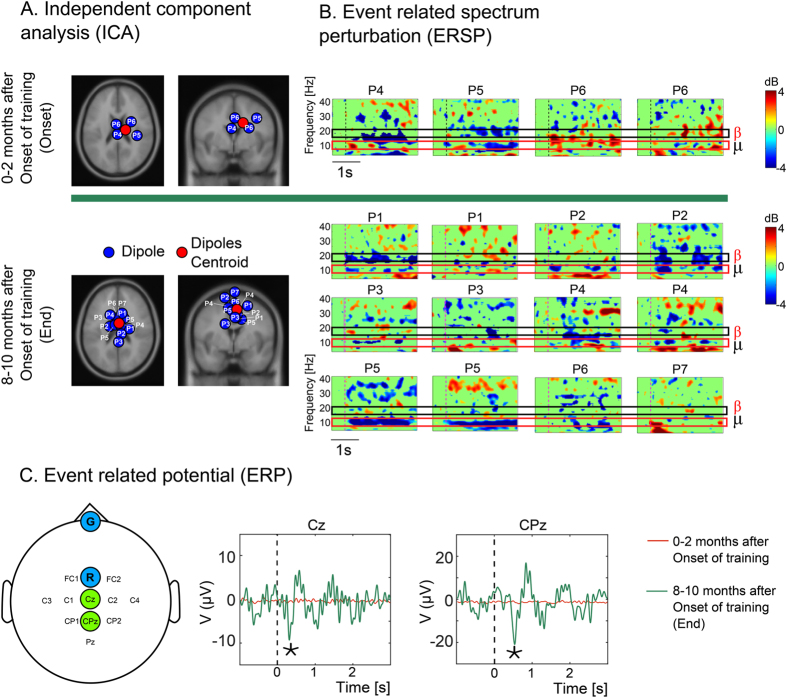
EEG recording. (**A**) Functional Cortical dynamics over the leg sensory motor area over time. Projection of the position of dipoles found by Independent Component Analysis during leg motor imagery. Analysis revealed that the number of dipoles observed in the S1/M1 cortex evolved from four at onset of the protocol to 12 at the end of training. Two sets of sessions are shown for patients 1 to 7: one recorded in the first 2 months of training (Onset) and one recorded between the 7^th^ and 9^th^ month of training (End). (**B**) For the onset and end of training, the Event Related Spectral Perturbation (ERSP) is shown for each of the Independent Components (IC) depicted in panel A. At the onset of training, one IC was found for P4 and P5, and two for patient P6. At the end of training, two ICs were found for each of the following patients: P1, P2, P3, P4 and P5. A single IC was identified for patient P6, and one for patient P7; and none for P8. Decrease in power in Beta waves (16·5–20 Hz) is associated with muscle contraction; suppression mu wave (7·5–12·5 Hz) are related to motor actions^46^. (**C**) Mean event related potential over all patients for two central electrodes (Cz and CPz) for Onset and End of training period. Significant desynchronization or synchronization is marked with an ‘*’.

At the onset of training, in three out of seven patients instructed to imagine moving their own legs, we could only identify a total of four significant independent components (sum for all patients) in the putative leg representation area of the primary somatosensory (S1) and motor (M1) cortices. The top shelf of Fig. 5A depicts the projection of these four components in the S1/M1 region (in a top and coronal slice) and the corresponding event related spectrogram perturbation (ERSP) found for each component is shown in Fig. 5B. The bottom shelf of Fig. 5A (respectively 5B for the ERSP) displays the same information, obtained after 8–10 months of training. Notice that at this point, a total of 12 significant components (sum for all patients) could be isolated in the leg area of the primary sensorimotor cortex of all seven patients who were asked to imagine locomotion movements.

Further analysis of the ERSP revealed the electrophysiological origins of the four independent components identified early in training in three patients (Fig. 5B, top shelf). They corresponded to the presence of desynchronization of beta waves (16–20 Hz) for Patients 4 and 5, a clear power reduction in mu rhythm (7–12 Hz, first panel left) in Patient 5, and a smaller desynchronization of mu in Patient 6.

In healthy subjects, mu rhythm desynchronization is observed during motor imagery^41^,^42^. Desynchronization of beta waves (16.5–20 Hz) is also observed during the preparation period, prior to the execution of voluntary limb movements^43^,^44^,^45^, as well as during motor imagery^46^.

The bottom shelf of Fig. 5B illustrates the changes that occurred in the ERSP after 8–10 months of training. At that point, a total of 12 Independent Components (IC) could be isolated in the leg representation area of the S1/M1 of all seven patients analyzed. In 4 out of 12 (P1 second IC, P2 first IC, P3 first IC and P7), we detected a reduction in mu power. Interestingly, the remaining eight ICs depicted a significant desynchronization in both mu and beta waves. Altogether, these findings indicate that, after prolonged BMI-based neurorehabilitation training, the leg representation area of S1/M1 cortices of all patients exhibited the type of desynchronization of beta wave which has been associated with motor imagery in healthy subjects^46^.

Further evidence for functional plasticity taking place over the training period was obtained through an event-related potential analysis, considering two central EEG electrodes, located on the leg representation area of the primary S1/M1 cortices, for all seven patients analyzed. Figure 5C shows that, at the onset of training, there was no significant desynchronization of the EEG (red line) when patients were asked to imagine walking. Eight to ten months later, however, a significant EEG desynchronization (green line) was observed when the average for all seven patients was considered.

According to our inclusion and exclusion criteria, all our patients exhibited a complete range of motion (ROM) of the joints and a maximal grade of lower limb spasticity of 2 on the Ashworth scale. As our training protocol progressed, we observed that all subjects maintained the original complete range of motion and did not develop any muscles contractures. Moreover, their level of lower limb spasticity did not increase their performance during the orthostatic or gait training. By using a Lokomat L-stiff test to quantify the level of spasticity of hip and knee muscles for flexors and extensors, we observed that, on average, all patients exhibited a reduced spasticity level by the end of 12 months. In addition, half of our patients maintained a stable bone mineral density index while the other half demonstrated a slight improvement. There was no correlation between bone mineral density and neurological improvement.

The SCIM questionnaire was applied to assess our patients’ level of functional independence to perform daily activities and mobility at home and in a community environment. Although all included patients exhibited a good level of independence in daily activities at the onset of training (ranging from 64 to 74 where scores ranged between 0 (dependent) to 100 (independent), some patients managed to improve their level of functional independence by the end of the training. For instance, two patients experienced an improvement in the frequency of bowel function with no need of using auxiliary devices (laxatives); two became more independent in the bathroom environment (perineal hygiene, setting towels/napkins and diapers); and two improved their level of independence in the process of transferring from the wheelchair to the toilet and one from the wheelchair to the car.

Overall, all patients ranked very high on emotional stability and obtained good scores on the quality of life, depression and self-esteem assessment, with minimal fluctuations throughout the study. According to individual demand, psychological support was increased, but no use of psychiatric medication was required.

## Discussion

As far as we can tell, this is the first clinical study to report the occurrence of consistent, reproducible, and significant partial neurological recovery in multiple chronic SCI patients. This partial recovery was manifested by improvements in both somatic sensations and voluntary motor control, below the level of the spinal cord lesions. This sensorimotor improvement was also paralleled by autonomic improvements, such as bowel function. Moreover, this is also the first report demonstrating partial clinical neurological improvements in SCI patients subjected to long-term training with a BMI-based gait protocol. Up to now, all previous clinical reports involving BMIs focused on decoding and control strategies of artificial prosthetic devices using the subject’s own electrical brain activity alone^12^,^13^,^14^,^47^,^48^. In these studies, patients were able to control the movements of artificial devices using their brain activity. Yet, none of these studies described any type of neurological recovery as a consequence of BMI training.

A total of eight chronic SCI patients were trained over the course of 12 months in a multi-stage, progressive neurorehabilitation protocol – the Walk Again Neurorehabilitation (WA-NR) protocol - that employed a non-invasive, EEG-based, closed-loop BMI approach. This protocol required that patients brain-control both virtual and mechanical actuators while receiving rich visuo-tactile feedback, aimed at restoring autonomous locomotion. Common to all periods of the WA-NR was the employment of both: (1) an EEG-based BMI, which required patients to produce motor imagery related to walking, was responsible for controlling the initiation of a series of lower limb motor behaviors (standing, walking and kicking a soccer ball), and (2) a multi-channel sensory substitution (remapping)^49^,^50^ strategy that utilized a haptic display applied to the skin surface of the patients’ forearms to deliver both tactile and proprioceptive-like feedback. Such real-time tactile/proprioceptive feedback of autonomous bipedal walking was combined with visual feedback (3) during physical training using a robotic Body Weight Support (BWS) system on a treadmill (LokomatPro), an overground BWS system (ZeroG), and a robotic exoskeleton.

For all our patients, clinical diagnosis of total (ASIA A) or partial (ASIA B) paralysis was confirmed, over multiple years, by routine clinical neurological examination, performed by different neurologists belonging to the clinical staff of the hospital in which these patients were followed. Previously, these patients were enrolled in a traditional physical rehabilitation program that mainly aimed at increasing independence in daily living activities, while seated in a wheelchair. Two patients (P2 and P6) had routine training in a standing orthostatic position (stand in table device). Six patients (P1, P3, P4, P5, P6, P7) had walking training using parallel bars or using a walker. None of these subjects exhibited any level of sensory or motor improvement or recovery in the many years they were followed prior to enrollment in our study.

At the onset of our protocol, the ASIA status of all eight patients was confirmed by our own initial neurological evaluation. That further supports our contention that the neurological improvement observed here resulted only from the new WA-NR introduced in the present study.

Overall, all eight patients involved in the study experienced a significant improvement in tactile, proprioceptive, vibration, and nociceptive (but not temperature) perception. Such improvement was already noticeable after 7 months, but reached its peak at the 10^th^ month of training. On average, such a sensory recovery spanned multiple dermatomes below the SCI level, being more vigorous and consistent for altered nociceptive perception (more than five dermatomes on average) than for tactile, vibration or proprioception (between one-two dermatomes). Thus, as a rule, the pattern of sensory recovery documented in all eight patients indicated a larger effect mediated by small myelinated or non-myelinated fibers, which normally convey nociceptive and high-threshold tactile information, than through the large myelinated fibers that normally mediate fine tactile discrimination and proprioception. This suggests that axons running through the spinothalamic tract were the main mediators of this somatosensory recovery. As such, this observation may imply that the spinothalamic tract may be more resistant to the initial SCI and/or remain more amenable than dorsal column-medial lemniscal fibers to underlie plastic recovery, even many years after a spinal cord lesion. Interestingly, this is consistent with previous studies in which somatosensory plasticity was documented in animals^51^,^52^.

It is important to mention that, although we have not documented any significant recovery in thermo sensation, this negative result may reflect primarily the lack of specificity of the clinical method employed to evaluate temperature sensing. In the future, we intend to repeat this analysis using a more sensitive technique.

In addition to significant sensory recovery, we also observed widespread improvement in voluntary muscle control below the level of SCI, even in patients clinically classified as having a complete SCI. Such a recovery in motor function, which progressed from proximal to distal muscles over time (Fig. 4A) and was more intense at the level of anti-gravitation (extensor) and flexor muscles involved in hip movements – despite the fact that some motor recovery was seen at the level of knee and even ankle joints - was corroborated by clinical examination, EMG recordings (Fig. 3B,C), and direct measurements of L-force generated by patients (Table 2). Such a pattern of motor recovery suggests mediation by intact fibers of the vestibulo-spinal tract (extensor muscles) that run in the ventrolateral portion of the spinal cord, next to the spino-thalamic tract. Motor recovery at flexor muscles suggests that some fibers of the rubro-spinal tract may have also remained intact in some of our patients.

Altogether, the partial neurological improvement observed meant that 50% of our patients could be reclassified (three from ASIA A to C and one from ASIA B to C) in less than a year of training with our neurorehabilitation protocol.

Prior to the present study, the literature contains only a single case report indicating that a patient with tetraplegia was reclassified from ASIA A to ASIA C after 3 years of being subjected to functional electrical stimulation bicycle therapy^53^. As far as we can tell, no independent study has reproduced this result so far. Heretofore, partial neurological recovery after an SCI has been mainly reported in subacute incomplete SCI patients. For instance, repetitive transcranial magnetic stimulation (rTMS), applied over the arm and leg representations of the primary somatosensory cortex of incomplete SCI patients, led to limited and variable improvements in sensory and motor functions^54^,^55^,^56^,^57^ primarily when high rTMS intensities were employed. Recently, the use of epidural stimulation at the lumbosacral level, combined with standing and stepping training, has allowed chronic ASIA A and B SCI patients to voluntary control paralyzed leg muscles. However, such motor control could only occur in the presence of the epidural stimulation^58^. In other words, these patients did not recover the ability to control their muscles without the assistive device. As such, none of the four subjects described by Angeli *et al*.^58^ changed their original ASIA classification. Interestingly, the study methodology also included a pre-implantation phase with extensive locomotor training (80 sessions) using a body weight support system on a treadmill. Neurological evaluations, neurophysiological measurements and ASIA exams, were performed before and after assisted gait training and implantation phases and no significant neurological recovery was observed suggesting that an isolated gait trainer with a BWS system on a treadmill does not produce meaningful neurological recovery in complete SCI patients. Moreover, no neurological recovery was described in a recent case report of a single SCI patient who was able to walk again using a BMI gait protocol that employed functional electrical stimulation of the lower limbs^6^.

Since our patients suffered their spinal cord lesions many years before enrolling in our protocol, the likelihood that the sensorimotor improvements observed here were due to spontaneous recovery can be basically ruled out. Indeed, a review by the International Campaign for Cures of Spinal Cord Injury Paralysis^59^ about spontaneous recovery after SCI, based on pharmaceutical clinical trials that focused on acute neuroprotection in SCI^60^,^61^,^62^, reported that the majority of spontaneous recovery occurs during the first 3 months after the SCI. Small residual clinical improvements can persist for up to 18 months, but only minor changes occur afterwards. Thus, 1 year after an SCI, 80% of the initial ASIA A cases remain A, about 10% convert to ASIA B and about 10% to ASIA C. A survey of 987 SCI patients showed that, between 1 and 5 years after the lesion, a conversion from ASIA A to a higher grade occurs in only 6.5% of patients (3.5% to B, 1.05% to C and 1.05% to D)^63^. Since some of the patients that moved from ASIA A to ASIA C in the present study had suffered their SCI more than a decade ago, it is highly unlikely that spontaneous recovery accounts for our findings.

In complete motor lesions (ASIA A and B), the majority of functional recovery occurs within the ZPP, following a craniocaudal sequence. Recovery within the ZPP appears to be due to both CNS and peripheral plasticity, while recovery beyond the ZPP would probably demand some CNS repair, likely involving axon regeneration^59^. Concerning partial motor recovery, the same review indicates that it likely occurs in myotomes with sensory preservation. Overall, the chances of a recovery of more than two spinal levels below the initial ASIA level are very small. Our findings revealed that motor recovery was indeed more significant within the ZPP. However, we also observed patients’ partial recovery in voluntary motor activity located in more than two dermatomes below the ZPP.

A previous study with ASIA B patients^64^ suggested that preservation of pinprick sensation can be useful in predicting motor recovery: presence of sacral pinprick sensation 4 weeks after the SCI significantly predicted ambulation 1 year later. Although these findings are not directly comparable to ours, by the differences in patient samples, the fact that we were able to document a 50% improvement in ASIA classification after many years of SCI raises the hypothesis that further motor clinical improvement could be seen with longer training.

The clear functional significance of the sensorimotor recovery observed here was further demonstrated by both the major overall improvement in the patients’ Walking Index. In other words, the observed partial sensorimotor recovery was translated into a meaningful improvement in the patients’ daily routine.

**Figure 6:**
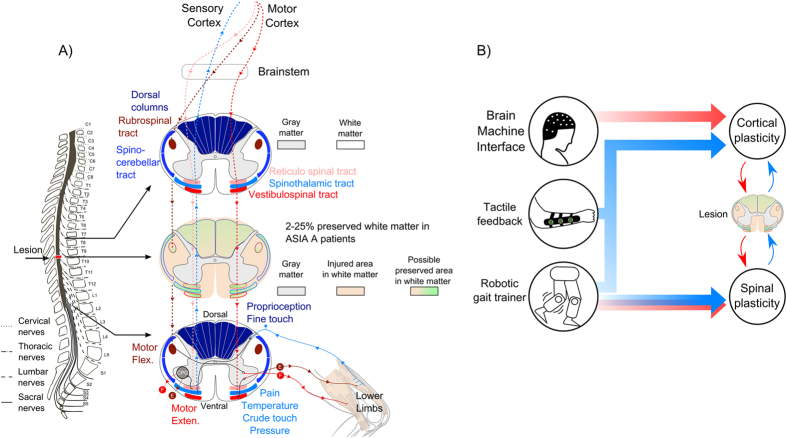
Hypothesis for mechanism of neurological improvement in SCI patients. **(A)** Example of spinal cord lesion in the thoracic area. Cross section is shown in the three parts of the spinal cord: at the lesion level, on top and under the lesion. Example of two efferent and one afferent pathways and their corresponding spinal tract are shown (rubrospinal and vestibulospinal tracts; spinothalamic tract respectively). At the lesion level we hypothesize preservation of 2–25% of white matter^18^ which might include the spinothalamic tract (sensory: pain, temperature, crude touch and pressure), vestibulospinal tract (motor: extensors muscles), rubrospinal tract (motor: flexors muscles) and dorsal columns (sensory: proprioception and fine touch). Under the lesion, Central Pattern Generators (CPGs) and its interaction with descending pathways (reticulospinal tract) and sensory afferents, modulating the gait pattern^50^. (**B**) Proposed components for the rehabilitation mechanism: direct brain control of virtual or robotic legs, continuous stream of tactile stimulation representing the missing haptic feedback from the legs and robotic actuators to train patients to walk. Cortical and spinal plasticity are hypothesized to change and to modulate neurological circuits in the preserved area around the lesion through motor (red) and sensory (blue) connections.

But what mechanism could account for this partial neurological recovery? Kakulas *et al*.^18^ showed that about 60% of SCI patients diagnosed clinically as having a complete spinal cord injury (ASIA A) still have 2–27% of the total area of spinal cord white matter preserved^18^. This finding is further supported by the observation that some of these surviving axons can exhibit functionality^17^ in more than 80% of such ASIA A patients. Sherwood and colleagues defined these cases as having a “discomplete” SCI and suggested that the residual axons could be functionally rescued to mediate some level of clinical recovery^17^. We propose that such a mechanism may have accounted for the partial neurological recovery observed in our study (Fig. 6A).

Assuming that residual spinal cord connectivity mediated the clinical recovery, what are the potential physiological mechanisms involved? Concomitantly to the partial neurological recovery, we documented the occurrence of cortical functional plasticity through longitudinal analysis of EEG recordings. This plasticity manifested itself by the emergence of consistent activation in the leg representation area of S1/M1, as measured by ICA, event-related spectrogram perturbation, and event-related potential analysis. The most prevalent EEG feature identified over training was the desynchronization of mu rhythm (7.5–12.5 Hz)^41^,^42^,^65^. Secondarily, we have also observed an increase in beta wave desynchronization.

Altogether, these results are consistent with our prior hypothesis^66^,^67^ that long-term BMI use is capable of changing the cortical body representation by including either new artificial actuators (robotic limbs or avatar bodies) or even by inducing a reactivation of the representation for paralyzed limbs, in the present case, legs. Therefore, based on these EEG findings, we propose that our long-term BMI-based training triggered a significant process of functional plasticity in S1/M1. Such functional cortical plasticity may have accounted for the re-emergence of lower limb representations in these cortical areas, as documented by the EEG analysis described above, and in another study with the same patients^50^. Such a functional cortical plasticity likely led to the reactivation of upper motor cortical neurons that normally project to the spinal cord, via the corticospinal tract. Given that a small fraction of spinothalamic, vestibulospinal and rubrospinal tract axons may have survived the initial SCI event and remained silent for many years, even in our ASIA A patients, the peculiar motor recovery observed in our study, involving primarily hip extensor and flexor muscles, could be explained by the functional reactivation of these residual axons as a byproduct of plasticity induced by long-term, intensive BMI training (Fig. 5).

But what are the key components of BMI training that triggered such a massive cortical plasticity? In our view, the driving force behind this plasticity includes: the direct brain control of robotic actuators by attentive and motivated patients, the reliance on patient’s motor imagery of walking to operate a BMI, a continuous stream of rich tactile/proprioceptive feedback, and the use of robotic actuators that allowed patients to routinely walk upright for long periods of time. This last feature may also have accounted for the generation of complex interactions between the supraspinal centers (cortical and subcortical structures) and the spinal cord.

Particularly, in the case of locomotion, the use of robotic gait training may have triggered the engagement of central pattern generators (CP), both at the supraspinal and spinal levels, and contributed to the generation of tactile and proprioceptive feedback from the patients’ own legs. These two components may have also induced functional plasticity at the spinal level and contributed to the type of sensory recovery observed below the level of the SCI, mediated mainly by the spinothalamic tract, and the somatosensory cortical plasticity described above.

In both animals^68^,^69^ and humans^70^, central pattern generators (CPGs) have been shown to generate bilateral rhythmic patterns, alternating motor activity between the flexor and extensor motoneurons in the absence of descending inputs^71^. Animal studies have shown that the gait pattern itself is generated in the spinal cord by CPGs, which are modulated by a peripheral sensory feedback. Conversely, once gait is re-established, proprioceptive and cutaneous afferents signals derived from receptors located in muscles, joints, and skin, can once again drive intraspinal circuits that interact with motor neurons, interneurons and CPGs in order to assist movement adaptations, such as postural corrections^69^. The level of this CPG activity would be determined by the brainstem locomotor command systems through the reticulospinal pathways^68^ (Fig. 5). The existence of CPGs in humans has been suggested, but its exact location is still unclear^71^.

Another possible source for sensory improvement observed in our patients could be the long-term use of visuo-tactile stimulation in virtual reality. Sensory modalities are not independent from each other; experiments have shown mechanisms of cross modal interaction^72^,^73^, and cross modal integration to create a robust perception^74^. In particular, vision of a body part was found to influence tactile perception^75^,^76^,^77^. For example, one experiment has shown that observation of a body part increases tactile acuity during passive touch in healthy subjects^78^. The effect was present when subjects were touched on their forearm while observing a different part of their own arm; and their accuracy was even increased when the body part was magnified^79^. No effect was observed if a neutral object was used in place of the arm. Patients with sensory deficiency (stroke patients) were found to improve their sensory resolution by observing their own body. In another experiment, magnifying the arm of a patient with chronic pain increased his/her pain, while minifying decreased it^80^. Similarly, we hypothesize that long-term training observing a human avatar mimicking the position and orientation of the patients’ body could have induced a positive effect on our patients’ sensory acuity.

Based on our clinical findings, we propose that long-term gait training with a BWS that employs BMI-based robotic actuators, combined with rich tactile feedback, could recruit the activation of CPGs in SCI patients^81^. Likely, BMI-based training and tactile feedback in a virtual reality environment could also enhance CPG activity by recruiting cortical afferents to influence locomotion control. If some corticospinal or vestibulospinal axons are still intact in a fraction of SCI patients, these locomotion-related signals could reach lower alpha-motor neurons below the level of the SCI. Moreover, by making patients walk routinely upright and against load, peripheral tactile and proprioceptive feedback would be generated and transmitted back to the spinal cord, contributing to the process of spinal cord functional reorganization (Fig. 6B).

In addition to partial neurological recovery, we observed a clear linear correlation between the number of hours spent upright walking/standing with the amount of bowel function (Fig. 4E). These and other autonomic improvements will be further explored in future studies using the same methodology.

We observed that significant clinical recovery was closely related to long-term and frequent use of a BMI paradigm that attempts to recreate lower limb movements in a realistic way (either in a virtual reality environment or through the use of brain-controlled robotic walkers). Further support for this contention can be found in the observation that after the two 30-day periods of vacation afforded to patients during the 12 month duration of the protocol, we noticed a reduction in sensory and motor capabilities below the SCI level (Figs 2 and 3). Such a temporary reduction was quickly reversed, however, by the restart of the BMI-based training protocol.

Introduction of rich tactile feedback signals, delivered via a haptic display, also seemed to have played a key role in the patient’s recovery. Experiments reported elsewhere have shown that patients are capable of incorporating an avatar body and extending their body schema toward the avatar legs employed in our training^50^. This suggests that the introduction of tactile feedback likely enhanced the ability of patients to exhibit cortical and/or subcortical functional plasticity during training with our BMI protocol, as we had previously documented in monkeys^67^,^82^.

Overall, the results obtained in our study suggest that BMI applications should be upgraded from merely a new type of assistive technology to help patients regain mobility, through the use of brain-controlled prosthetic devices, to a potentially new neurorehabilitation therapy, capable of inducing partial recovery of key neurological functions. Such a clinical potential was not anticipated by original BMI studies. Therefore, the present findings raise the relevance of BMI-based paradigms, regarding their impact on SCI patient rehabilitation. In this context, it would be very interesting to repeat the present study using a population of patients who suffered a SCI just a few months prior to the initiation of BMI training. We intend to pursue this line of inquiry next. Based on our findings, we anticipate that this population may exhibit even better levels of partial neurological recovery through the employment of our BMI protocol.

## Additional Information

**How to cite this article**: Donati, A. R. C. *et al*. Long-Term Training with a Brain-Machine Interface-Based Gait Protocol Induces Partial Neurological Recovery in Paraplegic Patients. *Sci. Rep.* **6**, 30383; doi: 10.1038/srep30383 (2016).

## Supplementary Material

Supplementary Information

Supplementary Movie S1

Supplementary Movie S2

Supplementary Movie S3

Supplementary Movie S4

Supplementary Movie S5

Supplementary Movie S6

Supplementary Movie S7

Supplementary Movie S8

Supplementary Movie S9

Supplementary Movie S10

Supplementary Movie S11

## Figures and Tables

**Table 1:** Patients’ demography.

	**Gen**	**Age**	**Lesion grade**	**Lesion level**		**Time since the lesion (years)**	**Etiology**
				**Right**	**Left**		
Patient 1	F	32	ASIA A	T11	T10	13	Closed trauma
Patient 2	M	26	ASIA B	T4	T4	6	Closed trauma
Patient 3	M	32	ASIA A	T10	T11	5	Open injury
Patient 4	M	38	ASIA A	T8	T8	5	Closed trauma
Patient 5	M	36	ASIA A	T7	T7	3	Closed trauma
Patient 6	M	29	ASIA A	T4	T4	8	Closed trauma
Patient 7	M	27	ASIA A	T7	T5	6	Closed trauma
Patient 8	F	29	ASIA A	T11	T11	11	Closed trauma

**Table 2:** Torque (N·m) measurement 7 and 12 months after beginning of training.

**Patient**	**Right Leg 7 months of training**	**Right Leg 12 months of training**	**Left Leg 7 months of training**	**Left Leg 12 months of training**
P1	17.7	21.14	16.85	16.12
P2	1.11	1.17	0.95	1.31
P3	2.76	4.42	4.34	4.6
P4	0.47	1.48	0.9	1.07
P5	0.93	4.3	1.22	2.13
P6	0.25	0.6	0.3	0.8
P7	0.45	0.89	0.58	1.2
P8	5.01		9.36	

Recordings were performed while patient was standing in the Lokomat (L-Force measurement). Patient was instructed by the physiotherapist to contract left or right leg (hip flexion). Peak of torque over six repetitions is reported.
