# Supplementary material for: Long-Term Training with a Brain-Machine Interface-Based Gait Protocol Induces Partial Neurological Recovery in Paraplegic Patients
Source: Sci Rep. 2016 Aug 11;6:30383. doi: 10.1038/srep30383 (PMC4980986; doi:10.1038/srep30383)
Supplement: Supplementary Information [file srep30383-s1.pdf]

SUPPLEMENTARY MATERIALS

LONG-TERM TRAINING WITH A BRAIN-MACHINE INTERFACE-BASED GAIT PROTOCOL INDUCES PARTIAL NEUROLOGICAL RECOVERY IN PARAPLEGIC PATIENTS

Ana R. C. Donati[1,2] , Solaiman Shokur[1], Edgard Morya[3,4], Debora S. F. Campos[1,2], Renan C. Moioli[3,4], Claudia M. Gitti[1,2], Patricia B. Augusto[1,2], Sandra Tripodi[1,2], Cristhiane G. Pires[1,2], Gislaine A. Pereira[1,2], Fabricio L. Brasil[3,4], Simone Gallo[5], Anthony A. Lin[1,10], Angelo K. Takigami[1], Maria A. Aratanha[3], Sanjay Joshi[6], Hannes Bleuler[5], Gordon Cheng[7], Alan Rudolph[8,10], Miguel A. L. Nicolelis[3,9,10,11,12]

[1] Neurorehabilitation Laboratory, Associação Alberto Santos Dumont para Apoio à Pesquisa (AASDAP), Sao Paulo, Brazil

[2] Associação de Assistência à Criança Deficiente (AACD), São Paulo, Brazil

[3] Edmond and Lily Safra International Institute of Neuroscience, Santos Dumont Institute, Macaiba, Brazil

[4] Alberto Santos Dumont Education and Research Institute, Sao Paulo, Brazil

[5] STI IMT, Ecole Polytechnique Federal de Lausanne, Lausanne, Switzerland

[6] Mechanical and Aerospace Engineering, University of California, Davis, CA, USA

[7] Institute for Cognitive Systems, Technical University of Munich (TUM), Munich, Germany, Germany

[8] Colorado State University, Fort Collins, CO, USA

[9] Department of Neurobiology, Duke University, Durham, NC, USA.

[10] Department of Biomedical Engineering, Duke University, Durham, NC, USA.

[11] Department of Psychology and Neuroscience, Duke University, Durham, NC, USA.

[12] Center for Neuroengineering, Duke University, Durham, NC, USA.

SUPPLEMENTARY METHODS

A. Inclusion / Exclusion Criteria

The inclusion criteria required patients to be either males or female subjects, ranging in age from 18-40 years, who incurred a spinal cord injury at least 1 year prior to initiation of the study. We included mainly subjects with a thoracic injury (paraplegia), with no comorbidities or offset comorbidities, and who were emotionally stable. To have a more accurate description of the clinical status each patient underwent the following tests: serum and urinary biochemical exams, kidneys/bladder ultrasonography, bone densitometry, and radiography of the spine, pelvis and lower limb. Five of eight patients underwent spinal fusion (arthrodesis) after the SCI, and the presence of orthopedic implants in an area of the body with no sensitivity is a contraindication for magnetic resonance imaging (MRI) to be performed (American College of Radiology). Exclusion criteria included: degree of spasticity > 2[1], degree of osteoporosis Tscore > -4,0 (World Health Organization), presence of joint deformities, fractures, peripheral neuropathy of the upper limbs, brain injury, or amputation of upper or lower limbs.

B. Training Routine

A multidisciplinary team composed of researchers, neuroscientists, engineers, biomedical engineers, physicians, nurses, physical therapists, and psychologists worked together during 12 months with the objective of proposing a new rehabilitation strategy, which integrated classic physical rehabilitation and long-term training with a brain machine interface (BMI) paradigm.

Eight subjects (two women and six men) with chronic spinal cord injury (3-13 years

of SCI), aged between 26-38 years, seven of them with complete injury (ASIA A) and one with incomplete injury (ASIA B), were included in an intensive gait training routine involving a BMI paradigm applied to both immersive virtual reality environments and robotic gait systems enriched with the ability to provide tactile feedback. The complexity of activities were increased over time, to ensure cardiovascular system stability and better postural control of the patients, starting with orthostatic training at stand-in-table device and progressing to robotic gait training, using a greater body-weight support (BWS) system on a treadmill (Lokomat)[2]. We then gradually decreased the BWS over time on a fixed overground track gait BWS system (ZeroG)[3]. A robotic exoskeleton was also used for the gait training. Clinical and BMI activities were integrated by proposing a multi-step BMI protocol, starting with training patients to use their EEG signals to interact with a brain-controlled 3D avatar, rendered in an immersive virtual reality setup. Next, patients learned to use the same EEG signals to control key locomotion movements generated by brain-controlled robotic gait devices (Lokomat and exoskeleton).

B1. Clinical Protocol

Clinical activities included traditional physical therapy with stretching and strengthening exercises and the use of a body-weight support (BWS) system for gait training: BWS ambulation on a treadmill and BWS ambulation on a fixed overground track[3].

Gait training using BWS on a treadmill system

The BWS used in combination with a treadmill system (Lokomat, Hocoma) included an electrically controlled gait orthosis composed of a hip support and two running orthotics, which allowed command of the hip and knee movements. Physiotherapists were able to control parameters such as the treadmill speed (1 to 2km/h), the body weight support (0 to 100%), the guidance force (0 to 100%) and the range of motion of the robotic joints (42-45° for hip flexion, 63-66° for knee flexion). Patients were also allowed to actively move the orthosis, in synergism with the computer-generated orthosis movements. The same equipment allowed us to assess spasticity, muscular strength during static and dynamic states, estimate active range of motion of the joints and distance walked. We fixed the range for BWS from 75-100%. Guidance force was fixed at 100% and the speed of the treadmill was set at 1km/h with the goal of promoting the safest possible training environment. Because subjects were not able to report on pain sensitivity, BWS was limited by the maximum knee extension without joint collapse during the stance phase of gait (knee collapse was observed about 75% of BWS, establishing a limit for further BWS reductions in this device). By using 100% of guidance force, the range of joint motion was adjusted by the physiotherapist who fixed the minimum and maximum values. That meant that joint control was maintained by the equipment. As guidance force was reduced and some of their weight was not supported directly by the BWS, subjects were in charge of adjusting their own posture.

Gait training using overground BWS system

The overground BWS system (ZeroG, Aretech LLC, Ashburn, VA) employed in our study uses a static or dynamic BWS while it rides along an overhead U-shaped fixed track. This device allows a greater freedom of movement and a better interaction between therapist and subject because there are no mechanical barriers among them. In this context, this equipment challenges the subjects more, by requiring postural control, trunk control, upper limb strength and dynamic balance during gait training. The device allows the control of BWS, speed, and provides a measurement of the distance walked. It also offers gait and balance training (static and dynamic) on different surfaces if necessary. The BWS contains an anti- fall system which provides greater security during all activities.

During gait training, subjects wore lower limb orthosis on both legs and a walking assistive device (hip-knee-ankle-foot orthosis [HKAFO] or ankle-foot orthosis [AFO] with knee extension splint and wheeled triangular walker). The following parameters were used during the session: range for BWS from 30 to 75%, fixed speed at 3km/h and fall distance between 5 and 10cm (which means that the displacement of the string at 5cm or 10cm triggers the anti-fall feature). Since patients started training with the Lokomat, achieving 75% of BWS, the ZeroG training started with 75%, progressing to 30% in some cases. The walking distance varied depending on the subject and on the subsequent sessions (individual upper limb strength and resistance, and cardiopulmonary performance).

B2. Brain Machine Interface / Tactile feedback / Virtual and robotic actuators

Brain Machine Interface (BMI) algorithm

A 16-channel EEG cap was used for all the experiments that involved the recording of cortical signals and their use in controlling virtual or robotic devices. In the initial phases of training, patients were instructed to imagine movements of their hands or arms. EEG electrodes were placed in order to maximize the recording area over the arm representation in the sensorimotor cortex. EEG patterns produced by this motor imagery were then decoded using a linear discriminant analysis (LDA), using features extracted by a 6-dimensional common spatial pattern (CSP) to construct an EEG classifier. Patients used the same motor imagery strategy to control both the simulated virtual avatar, the Lokomat, and the exoskeleton. They first selected a high level state of the actuator (for example 'walk' state) and then confirmed and triggered the execution of this motor command by performing an isometric contraction of the arm triceps. Using this simple strategy, patients could perform four primary commands 'stand' (except with the Lokomat), 'walk', 'stop', and 'kick'

A second control paradigm was introduced 7 months after onset of training. EEG signals were recorded over the leg sensorimotor cortex area. In this second strategy, subjects imagined moving their left and right legs to control the stepping of the ipsilateral legs. By alternating between left and right, stepping patients controlled the walking pattern of the avatar or the exoskeleton.

Generation and delivery of tactile feedback related to locomotion

The key objective of our strategy for tactile feedback was to provide SCI patients with key sensory information, lacking from their lower body, in a non-invasive way to

help generate the most realistic walking experience possible. During training in the virtual environment, virtual tactile signals were generated every time the avatar feet touched the ground. During training with robotic devices, artificial tactile information was generated by distance sensors placed on the patients immobilized legs and feet, in the case of the Lokomat, or in key locations of the exoskeleton, such as the plantar surface of the robotic feet. In both cases, contact with the ground generated a wave of pressure signals that could be delivered to the patients' forearm skin via a haptic display. By using this haptic display, all patients were able to sense the position of their legs in space and the contact of their (or the exoskeleton's) feet with the ground. In some experiments, the same arrangement was also employed to allow patients to experience the contact of the exoskeleton feet with a soccer ball during a "kicking" movement.

The haptic display employed vibrators (ERM vibrator consisting of a DC motor rotating an eccentric mass at different angular velocities) similar to the ones found in cellphones. Three vibrators spaced 6cm apart were integrated in the long sleeves of a shirt. These vibrators are coin-shaped and because of their small size (10mm x 2 mm) they can be easily integrated into a wearable tactile interface. This allowed the generation of various amplitudes and frequencies of vibration. ERM frequency and amplitude were coupled, and the maximum amplitude was reached at a frequency of 220 Hz which corresponds to the peak response frequency of Pacinian Corpuscles. Exploiting a haptic illusion called the 'Apparent Movement Illusion'[4,5], it was possible to produce the sensation of one continuous tactile feedback moving along the patients' forearm by sequentially triggering the three equidistant vibrators. While brain controlling the virtual avatar, the Lokomat or the exoskeleton, patients received tactile feedback

moving from their wrist to their elbow in synchrony with the rolling of the foot on the virtual or physical floor [6].

Virtual reality environment

Three virtual avatars (one female and two males) were designed and rendered using Autodesk Motion Builder (Autodesk 2014) software. Patients observed the avatar from a first person perspective using the Oculus Rift head mounted display (Oculus VR). The user could see the lower part of the avatar body in a position and orientation mimicking their own body. To increase their sense of immersion, rich visual (virtual stadium, various types of grounds) and auditory (sound of the stepping on the floor and background sound) elements were added.

Brain-controlled exoskeleton sensorized to deliver tactile feedback

A custom brain-controlled robotic exoskeleton (EXO) was designed for the execution of this project. To maximize power-to-weight ratio, this EXO employed electric motors and oil transmission hoses for a 12 degree of freedom actuation. The EXO was designed to be anatomically coherent with the body of our subjects. The hip-to-knee segments of the legs could be adjusted to accommodate a variety of different leg lengths. The EXO was stable in single support stance without the need of crouch, liberating the arms of the patients to execute any type of upper limb behavior. Patients could control the high level states of the EXO using EEG signals, while low level stabilization was done automatically by the robot.

Pressure sensors, wire sensors and gyroscopes were used for the PID controller of the exoskeleton to insure that the exoskeleton followed the correct trajectory.

Strain gauges and multimodal sensors[7] covered the exoskeleton's feet to detect forces exerted on the ground and confirm single stance and double stance positions. The information was conveyed to the patient through the tactile shirt.

C. Evaluation Metrics

C1. Medical Evaluation

The medical team provided integral support for the subjects during the research, performing clinical evaluations systematically before and after the activities to prevent and treat any possible complication. Due to the delicate pathological condition of the participants, greater attention was given to cardiovascular performance, skin inspection, spasticity and bowel and bladder emptying. Treatment for osteoporosis, a comorbidity presents in most of the subjects, was also instituted (alendronate sodium 70 mg and cholecalciferol 7000 IU, both weekly). Clinical evaluations were periodically used in order to identify possible changes in the neurological status of the spinal cord injury. For this purpose, the American Spinal Injury Association (ASIA) Impairment Scale (International Standards for the Neurological Classification of Spinal Cord Injury[8,9]) was employed as the main metric. Additional tests were used to supplement neurological sensory evaluation: the Semmes-Weinstein Monofilament Test[10], and clinical measurement of temperature, vibration, proprioception and deep pressure.

American Spinal Injury Association (ASIA) Impairment Scale

The ASIA evaluation test was applied five times during the 12 months of this protocol. Evaluation periods were organized in the following manner: (BI) Baseline - first evaluation after the injury, Month 0 - baseline of the present study in 2014, Month 4 -

period of intensive training, Month 7 - after 1 month break after 6 months of intense training, Month 10 - end of activities in 2014, Month 12 - early 2015, after the second break. The first clinical ASIA assessment was performed in each patient between 2 months and 3 years after the spinal cord injury. The baseline evaluation of the present study was performed 2 to 12 years after the first evaluation. During this period, the subjects were enrolled in a traditional physical rehabilitation program which yielded no sign of neurological recovery.

Patient name	Injury date	First ASIA recording after injury
P1	May 2001	Mar 2002
P2	Jul 2008	Jul 2009
P3	Apr 2009	Jul 2011
P4	Nov 2009	Sep 2010
P5	Oct 2011	Mar 2012
P6	Dec 2006	Jul 2009
P7	Sep 2008	Nov 2008
P8	Nov 2003	Nov 2004

Patient’s injury date and first ASIA recording after injury

To determinate the sensory level of the SCI we employed a pin-prick (pain sensitivity) in every dermatome in both left and right sides of the body. A comparison was made between the first stimulus done in the face (reference of normal sensitivity) and the second done in a thoracic dermatome or lower limb. The participant reported how he/she experienced the pain of the second stimulus: normal (Grade 2) or altered

sensitivity (hypoesthesia or hyperesthesia, Grade 1) or absence of sensitivity (Grade 0). To evaluate motricity the subjects were positioned in supine decubitus and a muscle function grading, varying from 0 to 5, was employed (Grade 0: absence of muscular activity; Grade 1: palpable or visible contraction; Grade 2: presence of muscle activity (active movement) throughout arch joint movement (full range of motion=ROM); Grade 3: presence of muscle activity, full ROM, against gravity; Grade 4: presence of muscle activity, full ROM, against gravity and with moderate resistance against an opposing force; and Grade 5: presence of muscle activity, full ROM, against gravity and with strong resistance against an opposing force.

All eight participants included in our study exhibited paraplegia; their upper limb muscles functioned normally (Grade 5). The lower limb muscles assessed were: the five key muscles (L2 hip flexors, L3 knee extensors, L4 ankle dorsiflexors, L5 long toe extensors, S1 ankle plantar flexors) and other abdominal and lower limb muscles (upper, medium and lower abdomen muscles, hip adductors, medial and lateral hamstring, gluteus maximus and medius, flexor and extensor digitorum longus muscles and long toe flexor). Because all subjects had a thoracic level SCI, the neurologic level of injury was defined according to the sensory evaluation.

ASIA Classification: ASIA A is characterized by absence of both motor and sensory functions in the lowest sacral area; ASIA B is defined by the presence of sensory functions below the neurological level of the SCI, including sacral segments S4-S5, and no motor function is preserved more than three levels below the motor level of the SCI on either side of the body; ASIA C by the presence of voluntary anal sphincter contraction, or sacral sensory sparing with sparing of motor function more than three

levels below the motor level, and the majority of key muscles have muscle grade less than 3. The ASIA allows even non-key muscle function more than three levels below the motor level to be used in determining motor incomplete status (B versus C); ASIA D is defined by presence of motor functions below the neurological level of the SCI and by at least half of key muscles below the neurological level having a muscle grade greater than or equal to 3; ASIA E is defined by normal sensory and motor functions (an individual without an initial SCI does not receive an ASIA grade).

Semmes-Weinstein Monofilament Test

This evaluation was done using nylon filaments of different thickness, distinguished by color and weight (blue 0.20g, purple 2.0g, red 4.0g, orange 10.0g, pink 300g). Originally used to test sensitivity in extremities (hand and foot), this test was applied to the trunk area in the present study, in order to expand and specify our tactile sensitivity evaluation. Each filament refers to a particular level of tactile sensitivity. As such, this test was used in order to better investigate multiple aspects of somatosensory sensation/discrimination. The monofilaments were used in a similar manner to the pin, by carrying out a comparison between the first stimulus done on the face (reference of normal sensitivity) and the second done in a thoracic dermatome or lower limb. The participant reported their perception of the second stimulus: normal, altered sensitivity, or absence of sensitivity.

Temperature, Pressure, Vibration, Proprioception

Temperature was evaluated using a dry cotton ball for warm sensation and an alcohol-soaked swab for cold sensation in every dermatome, on both sides of the body (right and left). Pressure pain (deep pain sensitivity) was assessed by using a

dynamometer (10g/mm2, maximum 8kg) applied to every dermatome (right and left sides). Vibration was evaluated using a diapason in a bone surface. The initial stimulation was delivered in an area proximal to the level of the SCI: upper limb (elbow) and upper trunk (third or fourth rib**)**. Then, the patient's perception was compared to the stimulation delivered to a distal area of the level of the injury. The sites that were stimulated were standardized in advance, but the sequence of the stimulation was performed in a random order involving the following locations: rib, hip (anterior superior iliac spine - ASIS), knee (patella), ankle (medial and lateral malleolus), calcaneus, hallux (head of first metatarsal) and sole of the foot. Patients were asked to describe where the stimulation was delivered (hip, knee, ankle, foot and, if possible, to specify which area of the foot was stimulated) and whether it was on the right or left side. For the proprioception evaluation, patients were asked to keep their eyes closed while the examiner did mobilization of the lower limbs. Patients were asked to describe the stimulation: which side of the body (right or left), which joint (hip, knee, ankle, toe), which movement (flexion, extension, adduction, abduction).

C2. Physical Therapy Evaluation

Our physical therapy and medical teams managed the physical training and applied the clinical evaluation during the study. They were also in charge of monitoring clinical conditions such as muscle strength (Lokomat L-force Evaluation[2,11]), trunk control (Thoracic-Lumbar Control Scale[12], gait performance (Walking Index Spinal Cord Injury II - WISCI second version[13]), level of independence (Spinal Cord Independence Measurement III - SCIM third version[14]), pain evaluation (McGill Pain Questionnaire)[15]

and Visual Analogic Scale (VAS)[16], spasticity (Modified Ashworth Scale and Lokomat L-stiff Evaluation[2,17]) and range of motion of lower limb joints (Medical Research Council Scale[18]).

Lokomat L-force Evaluation

In order to promote a more accurate quantitative analysis on muscle strength, we employed the L-force assessment tool, which is part of the Lokomat device. This tool was applied during three periods: beginning and end of the second half of 2014 and early 2015, after the second 1 month break. The L-force allows the evaluation of the maximum voluntary isometric muscle contraction (whose value is expressed in Newton meter) generated by the patient's flexor and extensor muscles of the hip and knee, while the patient was placed in a suspended resting static upright position with 30° of hip flexion and 45° of knee flexion, (with no weight bearing on the floor). While guided by the verbal commands of a physiotherapist, patients were asked to perform movements, including hip and knee flexion and extension, considering the right and left side individually. Each muscle group was assessed twice and the largest numerical value of two attempts was recorded.

Concomitant with the assessment of L-force, we also recorded electromyographic (EMG) activity generated by the lower limb musculature. A total of eight surface electrodes were used to capture EMG signals of four muscle groups in each leg. These included: rectus femoris proximal portion, gluteus maximus, medial hamstring and rectus femoris distal portion. The choice of these muscles was based on their motor functions studied by L-force: hip flexion (in this case, the most superficial muscles which assists in hip flexion corresponds to the proximal rectus femoris), hip

extension (gluteus maximus), knee flexion (medial hamstring is the muscle group of choice in these patients, because it is easier for its identification) and knee extension (distal rectus femoris).

Thoracic-Lumbar Control Scale

The Thoracic-Lumbar Control Scale was applied three times: twice in the second half of 2014 and again, after the 1 month break in December 2014, and at the beginning of 2015. This scale was developed in 2007 specifically for SCI patients. It evaluates quantitatively and selectively motor skill of the thoracolumbar region through 10 items in supine, prone, sitting and standing posture.

Walking Index Spinal Cord Injury II

The WISCI II is a revised version of the original measurement WISCI. It evaluates gait performance in SCI patients on a 10 meters route, based on the need to use assistive walking devices, braces, or physical assistance from a therapist. The rank ranges from 0, which means inability to keep a standing position, to 20, which refers to ambulation with no devices, no braces and no physical assistance. The measurement was applied 5 times (beginning and end of first semester of 2014, and three times in the second semester of 2014).

Level	Description
0	Client is unable to stand and/or participate in assisted walking.
1	Ambulates in parallel bars, with braces and physical assistance of two people, less than 10 meters.
2	Ambulates in parallel bars, with braces and physical assistance of two people, 10 meters.
3	Ambulates in parallel bars, with braces and physical assistance of one person, 10 meters.
4	Ambulates in parallel bars, no braces and physical assistance of one person, 10 meters.
5	Ambulates in parallel bars, with braces and no physical assistance, 10 meters.
6	Ambulates with walker, with braces and physical assistance of one person, 10 meters.
7	Ambulates with two crutches, with braces and physical assistance of one person, 10 meters.
8	Ambulates with walker, no braces and physical assistance of one person, 10 meters.
9	Ambulates with walker, with braces and no physical assistance, 10 meters.
10	Ambulates with one cane/crutch, with braces and physical assistance of one person, 10 meters.
11	Ambulates with two crutches, no braces and physical assistance of one person, 10 meters.
12	Ambulates with two crutches, with braces and no physical assistance, 10 meters.
13	Ambulates with walker, no braces and no physical assistance, 10 meters.
14	Ambulates with one cane/crutch, no braces and physical assistance of one person, 10 meters.
15	Ambulates with one cane/crutch, with braces and no physical assistance, 10 meters.
16	Ambulates with two crutches, no braces and no physical assistance, 10 meters.
17	Ambulates with no devices, no braces and physical assistance of one person, 10 meters.
18	Ambulates with no devices, with braces and no physical assistance, 10 meters.
19	Ambulates with one cane/crutch, no braces and no physical assistance, 10 meters.
20	Ambulates with no devices, no braces and no physical assistance, 10 meters.

Walking Index Spinal Cord Injury II - score

Spinal Cord Independence Measurement III (SCIM third version)

This test was used to evaluate the level of independence of the participants in daily activities and monitor their progress throughout the training. The scale was applied five times in 2014, three times in the first half and twice in the second half of the year. It classifies the ability to perform daily tasks, such as personal care, respiratory condition and sphincter control (bladder and bowel) and mobility in the home and community environment. The score ranges from 0 (totally dependent) to 100 (fully independent).

The McGill Pain Questionnaire / Visual Analogue Scale (VAS)

This is a self-report pain questionnaire that contains three sections. The evaluations were performed during five periods over the 12 months of training, in order to verify the existence, intensity, location and behavior of pain and how the patient was connected with it. The study of pain was important to guide its treatment when necessary and to assist in the investigation of neurological recovery. The Visual Analogue Scale is a simple assessment used to quantify pain. The patient is asked to draw a mark on a 10cm size horizontal line. The perception of pain increases on a scale that ranges from 0 to 10.

Medical Research Council Scale

This measurement has a component that quantitatively evaluates joint mobility (range of motion) passively, using a goniometer (CARCI®). The joints studied included the hip, knee and ankle, bilaterally, in the following movements: flexion/extension, abduction/adduction, internal/external rotation of the hip, flexion/extension of the knee, dorsi/plantar flexion of the ankle. This evaluation was performed during six periods over the 12 months of the study, in order to monitor the mobility during physical interventions.

Modified Ashworth Scale (MAS) and Lokomat L-stiff Evaluation

The Modified Ashworth Scale is based on a clinical assessment in which the examiner moves the patient's limb in order to quantify the increase in muscle tone (spasticity) in a 0-4 grade resistance movement (Grade 0: no increase in muscle tone; Grade 4: affected part rigid in flexion or extension). The assessed lower limb movements were flexion and extension of the knee and dorsiflexion and plantar flexion of the ankle, during six periods over the 12 months of training.

In order to make the assessment of spasticity more reliable, sensitive and with minimal external influence of the examiner, the Lokomat assessment tool L-Stiff was employed. This tool is related to the MAS and provides a quantitative measure of spasticity. The robotic device generates passive flexions and extensions of the hip and knee joints in the sagittal plane, in three different angular speeds: 30º/s, 90º/s and 120º/s[3]. The evaluation was conducted during three periods of the second half of 2014. The purpose of applying both instruments was to contribute to the treatment of spasticity and to monitor the physical safety of patients[7].

C3. Psychological Evaluation

Our psychology team provided continuous support to the patients during the entire 12 months of this protocol with the purpose of assessing emotional development and stability. Clinical measurements were also employed to assess quantitatively specific topics including: quality of life (WHOQoL-Bref - World Health Organization Quality of Life Assessment Instrument-Bref[19]); self-esteem (Rosenberg Self-Esteem

Scale); and depression (BDI - Beck Depression Inventory). The three tests were applied six times in 2014, in January, March, July, September, November and December.

The WHOQoL-Bref (World Health Organization Quality of Life Assessment Instrument-Bref) is a measurement validated for the population with traumatic etiology of Spinal Cord Injury. It presents a cross-cultural character and aims at evaluating the quality of life (QoL). The instrument consists of 26 questions, two of them related to a self-assessment of QoL and 24 questions divided in four aspects: physical, psychological, social relationships and environment. The answers were given on a Likert scale, whose score ranges from 1 to 5, according to the graduation of agreement or disagreement of the participant. There are no cut-off points to assess the instrument, so scores close to 0 indicate a poor quality of life and scores near 100 indicate a good quality of life[20].

The Beck Depression Inventory (BDI) is an instrument for measuring symptoms of depression. It consists of 21 items, four alternatives each, with scores ranging from 0 to 3, which imply increasing degrees of depression. The total score allows classifying the level of depression as minimal (score: 0 to 9), mild (score: 10 to 16), moderate (score: 17 to 29) and severe (score: 30 to 63)[21].

The Rosenberg Self-Esteem Scale (RSE) is a one-dimensional instrument that consists of 10 statements with four options that assess global self-esteem. The four possible answers are evaluated also using a Likert scale, ranging from “strongly agree” to “strongly disagree" and a score that ranges between 0 and 3. The final result allows the classification of self-esteem in low (score: 0 to 14), normal (score: 15 to 25) and high (score: 26 to 30)[22].

C4. EEG Analysis

EEG data analysis was performed with custom Matlab R2012a (The MathWorks Inc., MA) routines and EEGLAB 13.3.2b functions[23]. First, the raw EEG data was bandpass-filtered with a causal zerophase delay correction using a 1 Hz FIR high-pass filter (order 4000) and a 50 Hz FIR low-pass filter (order 36).

After that, we removed bad channels based on the kurtosis of data points from each channel using 5 standard deviations from the mean threshold limit (good channels present voltage values that are closer to a Gaussian distribution than noisy channels). Data was re-referenced to a common average reference calculated from the remaining EEG channels, and one channel was removed to avoid rank-deficiency in the Independent Component Analysis (ICA) algorithm. We then extracted -1 to 3s epochs from the continuous EEG data with respect to the event times. Bad epochs were identified using an amplitude threshold of -500 to 500 uV and a probability test with a 5 standard deviation from the mean threshold. In sequence, we decomposed the data into independent components (ICs) by performing an ICA using the JADE algorithm[24], followed by an equivalent dipole current source fitting procedure in which we only kept dipoles located within the brain and that had a maximum residual variance from the IC scalp projection of 15%; for this latter calculation, the DIPFIT EEGLAB toolbox[25] was used with a spherical head model in which each subject's electrode coordinates were aligned with the surface of a standard brain template. In order to cluster ICs from different subjects, we built a feature vector with their 5-25 Hz power spectrum, equivalent dipole position, and ICs scalp projection. The resulting vector dimension was reduced with a principal component analysis, and the first 10 principal components were

used as inputs to a k-means clustering algorithm. ICs that were located more than 3 standard deviations from the nearest cluster center were considered outliers and discarded. Clusters with components from less than half of subjects were discarded.

For each cluster and condition, we performed a series of measurements to highlight task-related brain dynamics modulations. Time-frequency maps revealing event related spectral perturbations (ERSPs) were calculated with respect to a baseline of 1s prior to the event and normalized by the average power across trials at each frequency; a 3-cycle Morlet wavelet was employed to obtain the frequency spectrum in every time window. Significant perturbations ($p<.05$) were determined using a non-parametric permutation method with 2000 surrogate data sets and this was subsequently used for masking the ERSP plots for significance. In addition, average event related potentials (ERPs) were determined for each subject in all conditions and submitted to a permutation statistical test for assessing significant effects of conditions in each channel ERP.

References

1. Bohannon, R. W. & Smith, M. B. Interrater reliability of a modified Ashworth scale of muscle spasticity. *Phys. Ther.* **67**, 206-207 (1987).
2. Lünenburger, L., Colombo, G., Riener, R. & Volker, D. in *IEEE 9th International Conference on Rehabilitation Robotics.* 345-348.
3. Hidler, J. *et al.* ZeroG: overground gait and balance training system. *J. Rehabil. Res. Dev.* **48**, 287-298 (2011).
4. Burtt, H. E. Tactual illusions of movement. *J. Exp. Psychol.* **2**, 371-385, doi:Doi 10.1037/H0074614 (1917).
5. Sherrick, C. E. & Rogers, R. Apparent Haptic Movement. *Percept. Psychophys.* **1**, 175-180, doi:Doi 10.3758/Bf03215780 (1966).

6. Shokur, S. et al. The walk again project: Sensory feedback for brain controlled exoskeleton. In: 44th Society for Neuroscience Meeting, 2014, Washington, D.C.. 44th Society for Neuroscience Meeting, Nov 15-19, (2014).

7. Mittendorfer, P. & Cheng, G. in *ROBOTIK 2012 7th German Conference on Robotiks.* 1-5 (VDE).

8. Ditunno, J. F., Jr., Young, W., Donovan, W. H. & Creasey, G. The international standards booklet for neurological and functional classification of spinal cord injury. American Spinal Injury Association. *Paraplegia* **32**, 70-80, doi:10.1038/sc.1994.13 (1994).

9. Maynard, F. M., Jr. *et al.* International Standards for Neurological and Functional Classification of Spinal Cord Injury. American Spinal Injury Association. *Spinal Cord* **35**, 266-274 (1997).

10. Bell-Krotoski, J. “Pocket filaments” and specifications for the semmes-weinstein monofilaments. *J. Hand Ther.* **3**, 26-31 (1990).

11. Bolliger, M., Banz, R., Dietz, V. & Lunenburger, L. Standardized voluntary force measurement in a lower extremity rehabilitation robot. *J Neuroeng Rehabil* **5**, 23, doi:10.1186/1743-0003-5-23 (2008).

12. Pastre, C. B. *et al.* Validation of the Brazilian version in Portuguese of the Thoracic-Lumbar Control Scale for spinal cord injury. *Spinal Cord* **49**, 1198-1202, doi:10.1038/sc.2011.86 (2011).

13. Morganti, B., Scivoletto, G., Ditunno, P., Ditunno, J. F. & Molinari, M. Walking index for spinal cord injury (WISCI): criterion validation. *Spinal Cord* **43**, 27-33, doi:10.1038/sj.sc.3101658 (2005).

14. Catz, A. *et al.* A multicenter international study on the Spinal Cord Independence Measure, version III: Rasch psychometric validation. *Spinal Cord* **45**, 275-291, doi:10.1038/sj.sc.3101960 (2007).

15. Melzack, R. The McGill pain questionnaire: from description to measurement. *Anesthesiology* **103**, 199-202 (2005).

16. Carlsson, A. M. Assessment of chronic pain. I. Aspects of the reliability and validity of the visual analogue scale. *Pain* **16**, 87-101 (1983).

17. Riener, R., Brunschweiler, A., Lünenburger, L. & Colombo, G. in *9th Annual Conference of the International FES Society.* p. 287-289.

18. Paternostro-Sluga, T. *et al.* Reliability and validity of the Medical Research Council (MRC) scale and a modified scale for testing muscle strength in patients with radial palsy. *J Rehabil Med* **40**, 665-671, doi:10.2340/16501977-0235 (2008).

19. Jang, Y., Hsieh, C. L., Wang, Y. H. & Wu, Y. H. A validity study of the WHOQOL-BREF assessment in persons with traumatic spinal cord injury. *Arch. Phys. Med. Rehabil.* **85**, 1890-1895 (2004).

20. Hill, M. R., Noonan, V. K., Sakakibara, B. M. & Miller, W. C. Quality of life instruments and definitions in individuals with spinal cord injury: a systematic review. *Spinal Cord* **48**, 438-450, doi:10.1038/sc.2009.164 (2010).

21. Beck, A. T., Ward, C. H., Mendelson, M., Mock, J. & Erbaugh, J. An inventory for measuring depression. *Arch. Gen. Psychiatry* **4**, 561-571 (1961).

22. Rosenberg, M. *Society and the adolescent self-image*. (Princeton University Press, 1965).

23. Delorme, A. & Makeig, S. EEGLAB: an open source toolbox for analysis of single-trial EEG dynamics including independent component analysis. *J. Neurosci. Methods* **134**, 9-21, doi:10.1016/j.jneumeth.2003.10.009 (2004).

24. Cardoso, J. F. High-order contrasts for independent component analysis. *Neural Comput.* **11**, 157-192 (1999).

25. Oostenveld, R. & Oostendorp, T. F. Validating the boundary element method for forward and inverse EEG computations in the presence of a hole in the skull. *Hum. Brain Mapp.* **17**, 179-192, doi:10.1002/hbm.10061 (2002).

SUPPLEMENTARY FIGURES

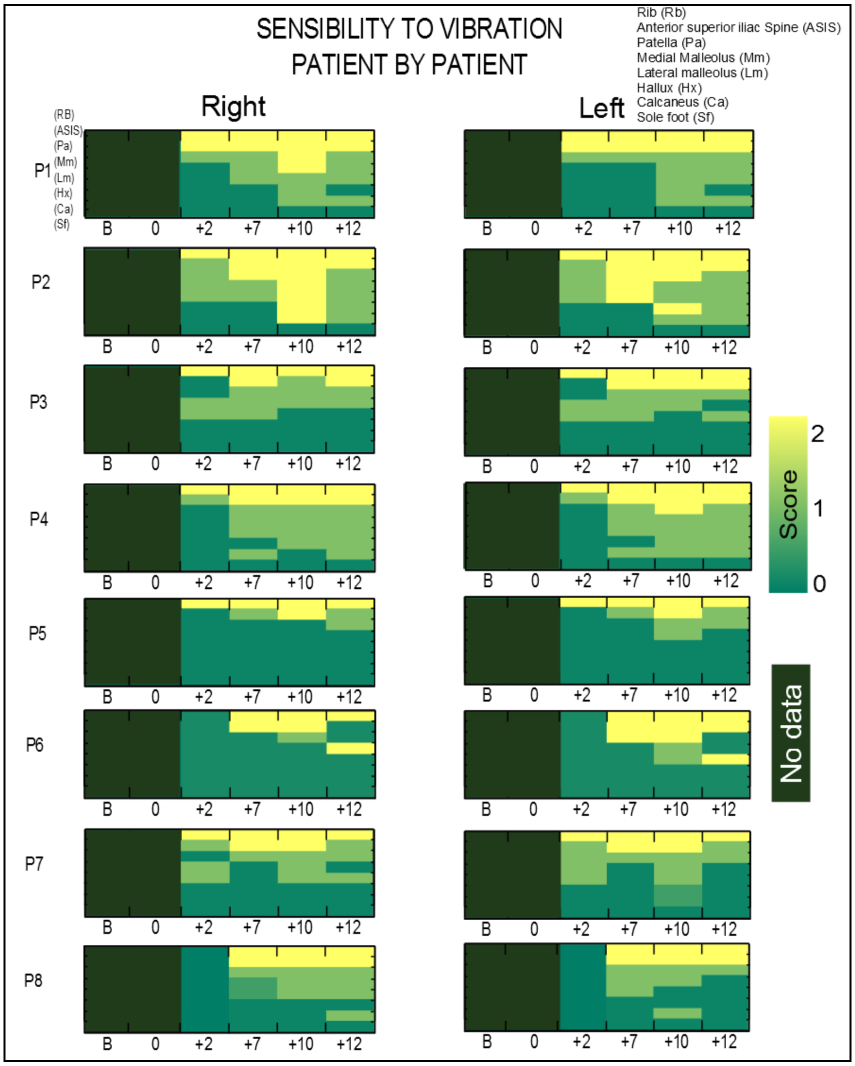

Figure S1. Details per patient of the sensitivity to vibration in the lower limbs. Patient's sensitivity to vibration on eight leg bones presented in a craniocaudal sequence. Score convention was the following: 0 for absence of sensation, 1 for altered sensation and 2 for normal sensation. The measurement was introduced at month 2 of the study. The patients were asked to compare a first stimulation performed in an area proximal to the SCI level with a second one performed below the SCI level. The sites that were stimulated were standardized, but the sequence of the stimulation was performed in a random order involving the rib, the hip

(ASIS), knee (patella), ankle (medial and lateral malleolus), calcaneus, hallux and sole foot. Patients were asked to describe where the stimulation was delivered (hip, knee, ankle, foot and, if possible, to specify which area of the foot was stimulated) and right or left side. Improvements were observed following a proximal to distal anatomic order and were more important at the hip joint for all patients, at some point of the study. All patients were able to distinguish stimulation delivered at the level of the ankle, in one or more evaluations. Best performance was observed at the ASIA B patient (P2).

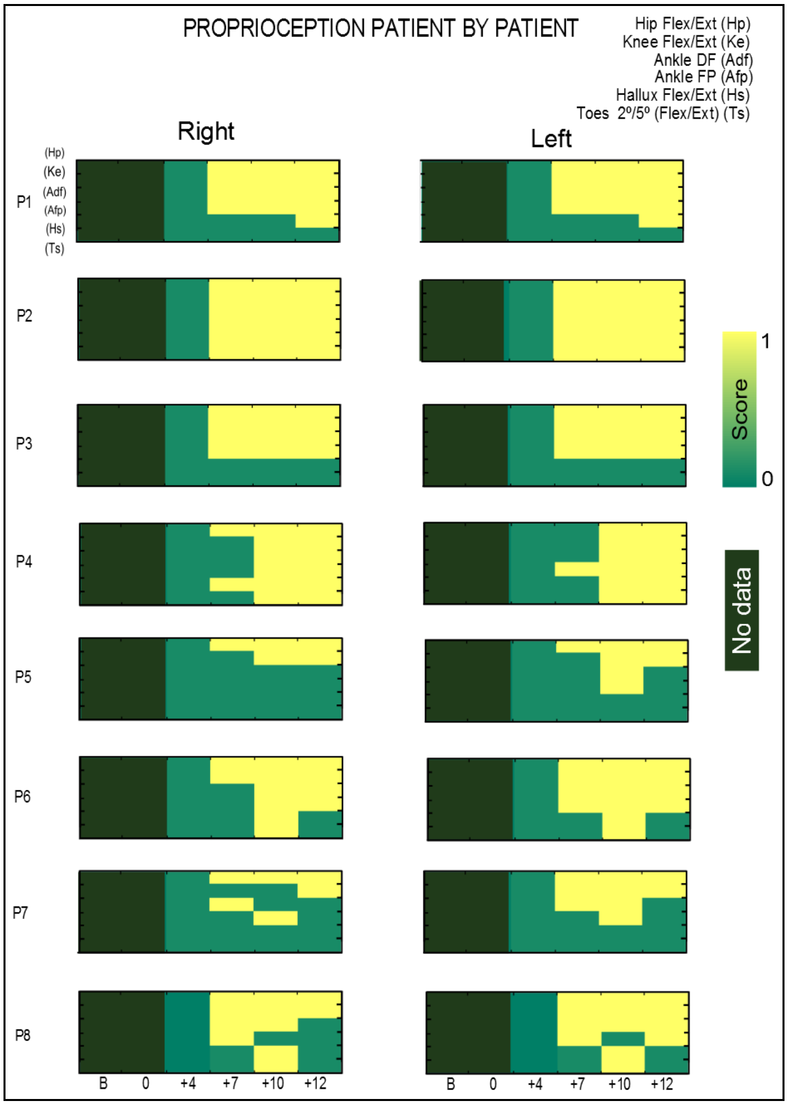

Figure S2. Score for lower limbs proprioception over all patients. Proprioception evaluation over lower limb joints (0: absent, 1: present). Measurement was introduced after 4 months of the onset of training. The examiner did mobilization of the lower limbs and the patients were asked to describe the stimulation: which side of the body (right or left), which joint (hip, knee, ankle, toe) and which movement (flexion, extension, adduction, abduction). From onset to 4 months of training no patient (including ASIA B patient, P2) could describe mobilizations performed in their lower limbs and nor could distinguish side of their body, that was being

stimulated. From the 4th to 7th month, patients exhibited better perception to this type of stimulation, mainly at the hip. P2 exhibited full recovery to describe lower limb mobilizations at the third evaluation (after 7 months of onset). Concerning ASIA A patients, better improvements were observed in subjects with lower levels of injury Patients 1, 3, and 8 (lower thoracic level of injury), followed by Patient 4 (medium thoracic level of injury) and Patient 5, Patient 7 (higher thoracic level of injury). An exception to this rule was observed in Patient 6, who exhibited an important performance at the evaluation, despite having higher thoracic level of injury.

Supplementary Movie Legends

Movie S1 - Sensory improvement over 12 months of training of Walk Again Neurorehabilitation paradigm (WA-NR). The average zone of preservation area is calculated over all patients per period. The zone of preservation designates the area where patients have some preserved altered sensation (hyper or hypoesthesia). Permission for publication granted by Associação Alberto Santos Dumont para Apoio à Pesquisa (AASDAP), Sao Paulo, Brazil.

Movie S2 - Motor improvement over 12 months of WA-NR protocol for 12 key and secondary lower limb muscles as shown in Figures 3A and 4A. Muscle transparency represents the motor score given by ASIA assessment. At baseline measurement, all muscles had a score of 0. Complete opaque muscle represents a score of 3 (contraction against gravity). Permission for publication granted by Associação Alberto Santos Dumont para Apoio à Pesquisa (AASDAP), Sao Paulo, Brazil.

Movie S3 - Lower limbs motor test in hanging position for patient P1 and P8 after 13 months of training with WA-NR protocol. Permission for publication granted by Associação Alberto Santos Dumont para Apoio à Pesquisa (AASDAP), Sao Paulo, Brazil.

Movie S4 - Lower limbs motor test in lying position for patient P1 after 13 months of training with WA-NR protocol. Permission for publication granted by Associação Alberto Santos Dumont para Apoio à Pesquisa (AASDAP), Sao Paulo, Brazil.

Movie S5 - Lower limbs motor test in lying position for patient P8 after 13 months of training with WA-NR protocol. Permission for publication granted by Associação Alberto Santos Dumont para Apoio à Pesquisa (AASDAP), Sao Paulo, Brazil.

Movie S6 - Lower limbs motor test in lying position for patient P3 after 13 months of training with WA-NR protocol. Permission for publication granted by Associação Alberto Santos Dumont para Apoio à Pesquisa (AASDAP), Sao Paulo, Brazil.

Movie S7 - Lower limbs motor test in lying position for patient P5 after 13 months of training with WA-NR protocol. Permission for publication granted by Associação Alberto Santos Dumont para Apoio à Pesquisa (AASDAP), Sao Paulo, Brazil.

Movie S8 - Lower limbs motor test in the lokomat (turned off) for patient P1 after 13 months of training with WA-NR protocol. Permission for publication granted by Associação Alberto Santos Dumont para Apoio à Pesquisa (AASDAP), Sao Paulo, Brazil.

Movie S9 - Lower limbs motor test in hanging position for patient P1 after 9 months of training with WA-NR protocol. Permission for publication granted by Associação Alberto Santos Dumont para Apoio à Pesquisa (AASDAP), Sao Paulo, Brazil.

Movie S10 - Lower limbs motor test in hanging position for patient P8 after 13 months of training with WA-NR protocol. Permission for publication granted by Associação Alberto Santos Dumont para Apoio à Pesquisa (AASDAP), Sao Paulo, Brazil.

Movie S11 - Patient P2 performing a brain control exoskeleton walk. Permission for publication granted by Associação Alberto Santos Dumont para Apoio à Pesquisa (AASDAP), Sao Paulo, Brazil.
